# UBE2E2 enhances Snail-mediated epithelial-mesenchymal transition and Nrf2-mediated antioxidant activity in ovarian cancer

**DOI:** 10.1038/s41419-023-05636-z

**Published:** 2023-02-10

**Authors:** Xiaoling Hong, Ning Ma, Danjie Li, Mengwen Zhang, Wenqiuzi Dong, Jie Huang, Xinxin Ci, Songling Zhang

**Affiliations:** 1 Department of Obstetrics and Gynecology, The First Hospital of Jilin University, Changchun, 130021 Jilin China; 2 Institute of Translational Medicine, The First Hospital of Jilin University, Changchun, 130021 Jilin China

**Keywords:** Ovarian cancer, Metastasis, Ovarian cancer

## Abstract

Dissemination of ovarian cancer (OvCa) cells can lead to inoperable metastatic lesions in the bowel and omentum, which have a poor prognosis despite surgical and chemotherapeutical options. A better understanding of the mechanisms underlying metastasis is urgently needed. In this study, bioinformatics analyses revealed that UBE2E2, a less-studied ubiquitin (Ub)-conjugating enzyme (E2), was upregulated in OvCa and was associated with poor prognosis. Subsequently, we performed western blot analysis and IHC staining with 88 OvCa and 26 normal ovarian tissue samples, which further confirmed that UBE2E2 protein is highly expressed in OvCa tissue but weakly expressed in normal tissue. Furthermore, the silencing of UBE2E2 blocked OvCa cell migration, epithelial-mesenchymal transition (EMT) and metastasis in vitro, whereas UBE2E2 overexpression exerted the opposite effects. Mechanistically, UBE2E2 promoted p62 accumulation and increased the activity of the Nrf2-antioxidant response element (ARE) system, which ultimately activated the Snail signaling pathway by inhibiting the ubiquitin-mediated degradation of Snail. Additionally, co-IP and immunofluorescence demonstrated that a direct interaction exists between UBE2E2 and Nrf2, and the N-terminal of UBE2E2 (residues 1-52) is required and sufficient for its interaction with Nrf2 protein. Mutations in the active site cysteine (Cys139) impaired both the function and cellular distribution of UBE2E2. More importantly, the deletion of UBE2E2 reduced tumorigenicity and metastasis in xenograft OvCa mouse models. Taken together, our findings reveal the role of the UBE2E2-Nrf2-p62-Snail signaling axis in OvCa and thus provides novel therapeutic targets for the prevention of OvCa metastasis.

## Introduction

Ovarian cancer (OvCa) is the most aggressive and lethal gynecologic malignancy [1] and is characterized by the propensity for OvCa cells to “slough off” from the primary tumor site and establish metastases. In 2021, OvCa was the fifth leading cause of cancer deaths in women in the USA [2]. Approximately 75% of patients with OvCa are diagnosed at an advanced stage, when radical cytoreductive surgery and chemotherapy are less effective, and the 5-year overall survival (OS) rate has dropped to 29%, whereas patients with early-stage disease have an OS of `90% [3]. The challenge is to develop novel strategies to improve the early diagnosis of OvCa and the treatment efficacy for patients with metastasis. Therefore, it is necessary to dissect the cellular and molecular mechanisms underlying the high invasiveness of OvCa.

In contrast to other human malignancies that metastasize through the hematogenous spread of tumor cells, OvCa cells can directly migrate into the peritoneal cavity via peritoneal fluid which makes the omentum the most common site of OvCa metastasis. Epithelial-mesenchymal transition (EMT), a major mechanism involved in the initiation of tumor metastasis, involves a wide range of phenotypic and molecular changes leading to cancer cells losing their epithelial characteristics and dedifferentiating into mesenchymal-like cancer stem cells (CSCs) [4]. The zinc finger protein SNAI1 (also known as Snail; UniProt/Swiss-Prot: O95863, gene name *SNAI1*), represses the expression of a broad repertoire of epithelial genes, including *E-cadherin/CDH1*, and is a master regulator of EMT in most cancers [5]. Snail is a highly unstable protein that is rapidly degraded by the proteasome. In addition, many other key factors critical for EMT, such as Slug/SNAI2, Twist1 and Zeb2, are also regulated by the ubiquitin (Ub) proteasome system (UPS) [6]. The scaffold protein p62 (sequestosome 1 or SQSTM1), an adaptor that connects ubiquitination with autophagy, was recently reported to be associated with the ubiquitination and proteasomal degradation of the Snail protein in various disease models, such as glioblastoma [7], bladder tumor [8], and cardiac fibrosis [9]. However, whether p62 influences the ubiquitination and expression level of Snail in OvCa remains unknown.

Ubiquitination, a posttranslational modification, plays a fundamental role in degrading proteins and regulating most cellular processes [10]. Ubiquitination is executed through an enzymatic cascade consisting of Ub-activating enzymes (E1), Ub-conjugating enzymes (E2), and Ub protein ligases (E3). In addition to interacting specifically with and discriminating between Ub and ubiquitin-like proteins (UBLs), E2s are scaffolding proteins/noncovalent regulators in processes that are independent of their enzymatic activity [11]. Canonical E2s can consist exclusively of the catalytic core (ubiquitin-conjugating/UBC) domain (class I) and contain COOH- (class II) or NH2- terminal extensions (class III) or both (class IV) [12]. Through gene expression and functional annotation analyses, we identified the Ub-conjugating enzyme E2E2 (UBE2E2) with upregulated expression in OvCa and linked it with detrimental outcomes in patients with OvCa. UBE2E2 (also called UbcH8) was first reported in 1997 and has been proposed to play a pathological role in various human diseases, including type 2 diabetes (T2D), rheumatic autoimmune disease, Parkinson’s disease, non-small cell lung cancer, hepatocellular adenoma and carcinoma [13–15]. In addition, UBE2E2 has been identified in relation to adipogenesis and RR intervals [16]. Nevertheless, most of the evidence is based on genome-wide association studies (GWAS) and meta-analyses, and therefore, the molecular function of UBE2E2 remains poorly characterized.

In this study, we observed that UBE2E2 promoted OvCa cell migration and EMT by upregulating Snail expression and downregulating E-cadherin expression. In addition, UBE2E2 enhanced the overall cellular antioxidant capacity by regulating the stability and activity of the antioxidant transcription factor, nuclear factor-erythroid-2 related factor-2 (Nrf2). P62 was found to be accumulated during UBE2E2-induced EMT, and this accumulation led to activation of the Snail signaling pathways. Herein, our data demonstrate that UBE2E2 plays tumor-promoting roles in OvCa and suggest its potential use in a novel therapeutic strategy.

## Materials and methods

### Cell lines and compound preparation

Human OvCa A2780 and SKOV3 cell lines were obtained from ECACC and ATCC, respectively, and authenticated by Genetic Testing Biotechnology Corporation (Suzhou, China) by detecting short tandem repeat (STR) markers. All cell lines were grown in RPMI-1640 medium supplemented with 10% fetal bovine serum (Gibco, USA) and 1% penicillin/streptomycin (TransGen Biotech, China) in incubators with 5% CO_2_.

A2780 UBE2E2-knockout cells were created using CRISPR/Cas9 technology. Plasmids expressing Cas9, GFP, puromycin resistance markers, and single guide RNAs (sgRNA) targeting exon 2 of human *UBE2E2* (the sequences are shown in Fig. 2A) were cotransfected into A2780 cells. The transfected cells were enriched by selection with puromycin (2 µg/ml) and FACS, and clones were selected by dilution. The loss of UBE2E2 expression was verified by qRT-PCR and western blot analysis.

MG132 and t-butylhydroquinone (tBHQ) (MedChem Express, USA) were dissolved in dimethyl sulfoxide (DMSO) and maintained at −20 °C.

### Patient sample collection

In this study, a total of 88 OvCa specimens and 26 normal ovarian tissue specimens were obtained from the Department of Obstetrics and Gynecology at the First Hospital of Jilin University. Informed consent was obtained from all the patients undergoing surgical resection, and the study was approved by the Ethics Committees of the hospital. All tumor samples were diagnosed as high-grade serous ovarian carcinoma, and all normal ovarian tissues were confirmed to be free of tumors via histological analysis. Subsequently, tissue sections were fixed and immunostained with anti-UBE2E2 and anti-β-actin antibodies.

### Cell transfection

Short interfering RNA (siRNA) duplexes specifically targeting UBE2E2 were synthesized by Hanbio (Shanghai, China); the sequences are listed in Table S1. These siRNA duplexes and transfection reagent (Hanbio, China) were mixed separately with Opti-MEM (Gibco, USA), and the two mixtures were combined and incubated at room temperature (RT) for 10 min. The transfection mixture was then added to the cell culture medium, and the cells were analyzed 72 h after transfection.

Recombinant adenovirus vectors containing the mRFP-GFP-LC3 reporter were purchased from Hanbio (Shanghai, China) and introduced into cells according to the manufacturer’s instructions. The full coding sequence and truncation mutants of UBE2E2 were subcloned into pEGFP-C1-Flag expression vectors. Full-length p62 and Snail were subcloned into pCMV expression vectors, and full-length Nrf2 was subcloned into the VR1012 vector.

### Cell proliferation and colony formation assays

For the assessment of viable cell proliferation, 2 × 10^4^ A2780 cells were seeded in 24-well plates, and at the indicated time points, the cells were subjected to trypan blue staining and counted using a hemocytometer. SKOV3 cells were seeded (5 × 10^4^ cells/well) 24 h after siRNA transfection.

For the assessment of colony formation, SKOV3 cells were seeded 24 h after siRNA transfection. The cells were plated in 6-well plates (800 cells/well), and 10 days later, the colonies were stained, photographed and counted.

### Wound healing assays

Cells were seeded in 12-well plates and cultured until they reached complete confluence. Thin, straight wounds were then scratched with 200-μl pipette tips. After washing with PBS, the medium was replaced with fresh serum-free medium (to prevent cell proliferation). The wounds were photographed under an inverted microscope 0 and 24 h after scratching, and the gaps between the cells were evaluated using ImageJ software. The ratio of migration was calculated as follows: ([gap width at 0 h] − [gap width at 24 h]) / (gap width at 0 h).

### Transwell assays

Approximately 2 × 10^4^ cells were seeded in serum-free medium onto upper Transwell chambers (Costar, Corning, USA) containing a polycarbonic membrane (diameter of 6.5 mm and pore size of 8 μm). In addition, 700 μl of RPMI-1640 medium supplemented with 20% serum was added to the lower chamber as a chemoattractant. After 48 h of incubation at 37 °C, the invasive cells located on the lower surface of the membrane were stained with 0.02 g/ml crystal violet solution and counted.

### Western blot analysis and coimmunoprecipitation (co-IP)

Cell lysates were prepared in lysis buffer (Beyotime Biotechnology) and 1× loading buffer (TransGen Biotech). The samples were separated by 12% SDS–PAGE (Bio-Rad) and transferred onto PVDF membranes (Immobilon-FL, Millipore) for subsequent immunoblotting. Protein expression levels were detected with specific primary antibodies and goat anti-rabbit IgG and goat anti-mouse IgG secondary antibodies (Jackson ImmunoResearch Laboratories). Anti-Snail, anti-vimentin, anti-β-actin, anti-Flag, anti-p27 mouse monoclonal antibodies and anti-UBE2E2, anti-Slug, anti-N-cadherin, anti-E-cadherin, anti-p62, anti-Ub, anti-β-tubulin, anti-Lamin B1 rabbit polyclonal antibodies were purchased from Proteintech Group, Inc. (Chicago, USA). Anti-Nrf2, anti-HO-1, anti-NQO1, anti-GCLC, anti-GCLM, and anti-LC3B rabbit polyclonal antibodies were purchased from Cell Signaling Technology (CST, USA). HRP-conjugated secondary antibodies were visualized with an enhanced chemiluminescence (ECL) system (Millipore, USA) and analyzed with Image Lab 3.0 (Bio-Rad).

Co-immunoprecipitation (co-IP) was performed using a Flag-tagged protein IP assay kit with magnetic beads (Beyotime Biotechnology, China) according to the manufacturer’s instructions.

Nuclear-Cytosol Extraction kit (Beyotime Biotechnology, China) was used for the isolation of cell components according to the manufacturer’s instructions.

### RNA extraction and quantitative RT-PCR (qRT-PCR)

Total RNA was extracted from cultured cells with an RNA extraction kit (TransGen Biotech, China) according to the recommended protocol. After quantification, cDNA was reverse transcribed from 1 μg of total RNA with a First-Strand cDNA Synthesis SuperMix kit (TransGen Biotech). Real-time qPCR was carried out using a StepOne Real-Time PCR system (Applied Biosystems) with LightCycler SYBR Green (Roche). The primers used are listed in Table S2. All samples were run in triplicate. The relative expression of mRNA was calculated according to the 2^−ΔΔCt^ method after normalization to GAPDH expression.

### Immunohistochemical (IHC) staining

Tissue sections were deparaffinized, rehydrated and then incubated with 3% H_2_O_2_ in methanol for 20 min at RT. Antigen retrieval was performed by heating for 20 min at 95 °C in 0.01 M sodium citrate buffer (pH 6.0). The slides were then incubated overnight with primary antibody at 4 °C. After incubation with the secondary antibody for 1 h, immunostaining was visualized with DAB, and the slides were counterstained with hematoxylin. The IHC score was determined by adding the staining intensity (scored as 0, negative; 1, light yellow, weakly positive; 2, yellowish brown, moderately positive; and 3, brown, strongly positive) and the percentage of positive cells (scored as 1, ≥0% to <25%; 2, ≥25% to <50%; 3, ≥51% to <75%; and 4, ≥75%).

### Immunofluorescence

Cells were seeded on glass plates and transfected with plasmids or treated with 50 µM tBHQ for 24 h. After fixation in 4% paraformaldehyde in PBS for 30 min, the cells were permeabilized with 0.2% Triton X-100 for 10 min, blocked with goat serum for 1 h, and incubated overnight with anti-p62 or anti-Nrf2 antibody at 1:500 dilution. Alexa Fluor 594-conjugated goat anti-mouse IgG (Life Technologies) was used as the secondary antibody. The nuclei were counterstained with DAPI (10 µg/ml). Images were captured using an inverted epifluorescence microscope (Olympus).

### Xenograft OvCa mouse models

Six- to 7-week-old female BALB/c nude mice were obtained from Beijing Vital River Laboratory Animal Technology Co., Ltd. (China) and randomly assigned to two groups. To generate orthotopic xenograft models for OvCa, ~2 × 10^5^ cells (control cells or UBE2E2-knockout cells, in 20 μl cell suspension) were gently transplanted into the ovarian bursa area in each mouse (*n* = 8 for each group). To generate the intraperitoneal xenograft models, ~5 × 10^6^ cells (control cells or UBE2E2-knockout cells) were intraperitoneally injected into each mouse (*n* = 8 for each group). After 40 days, all the mice were sacrificed, and fluorescence imaging was performed using an in vivo imaging system (IVIS lumina III, PerkinElmer). In addition, the tumor tissues were dissected, photographed, and prepared for western blotting or immunostaining. All experimental protocols were conducted in accordance with the guidelines of the Animal Research Committee of Jilin University.

### Statistical analysis

All statistical analyses were performed using GraphPad Prism 6 (GraphPad Software, USA) and are presented as the means ± standard errors of the means (SEMs). Student’s *t* tests were used for the comparison of parameters between two groups. Differences with *p* values < 0.05 were considered to be statistically significant.

## Results

### Upregulation of UBE2E2 expression is associated with poor prognosis for OvCa patients

High-grade serous ovarian cancer (HGSOC) is the most common type of OvCa, accounting for 75% of all epithelial OvCa. An analysis of the gene expression data from The Cancer Genome Atlas (TCGA) and Genotype-Tissue Expression (GTEx) database identified 7638 genes as differentially expressed genes (DEGs) based on the ovarian serous cystadenocarcinoma and normal ovary sample gene expression profiles. Among these DEGs, 27 genes were found to be related to ubiquitin-mediated proteolysis according to a KEGG pathway analysis. Within this select group, the expression of 7 genes was upregulated in OvCa, and these DEGs were associated with detrimental clinical outcomes of patients. We then focused on UBE2E2, whose function in ovarian carcinogenesis had not been explored (Fig. S1).

**Fig. 1:**
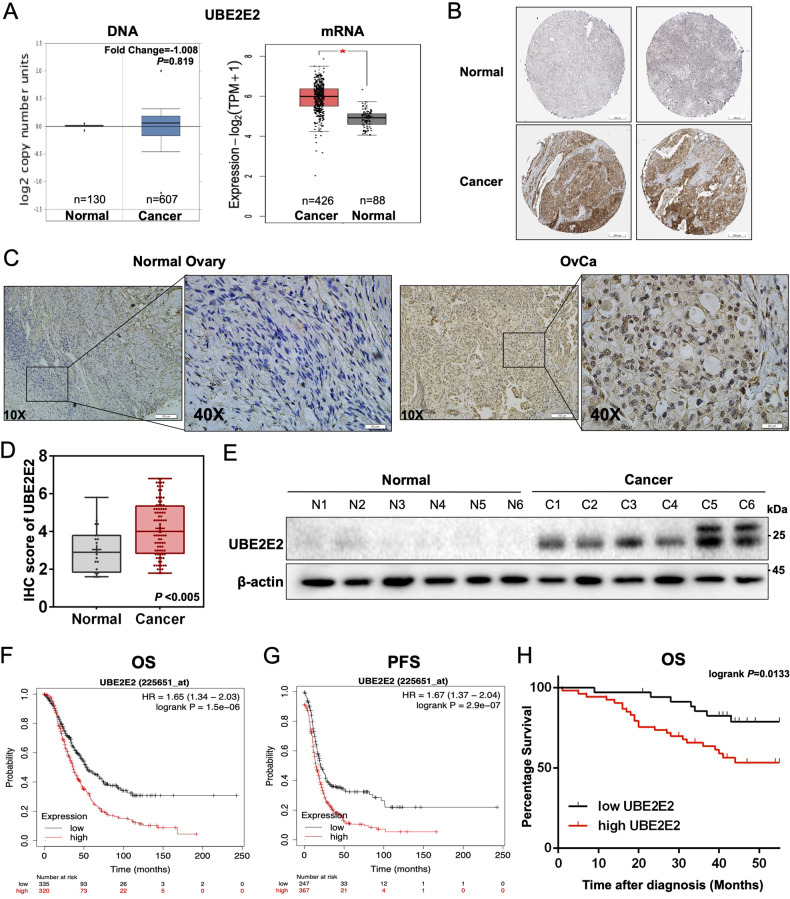
UBE2E2 is upregulated and associated with poor prognosis in OvCa. **A**, **B** The gene expression profile data were obtained from the GEPIA (http://gepia2.cancer-pku.cn/), Oncomine (https://www.oncomine.org/) and The Human Protein Atlas (https://www.proteinatlas.org/) (antibody: HPA028872) online databases. **C** Representative images of the immunostained UBE2E2 in two patient samples (×10 magnification, scale bar = 100 μm; ×40 magnification, scale bar = 20 μm) are shown. **D** The protein level of UBE2E2 was significantly increased in OvCa tissues (normal ovarian tissue, *n* = 20; OvCa tissue, *n* = 88). **E** Western blot analysis was performed to determine the expression levels of UBE2E2 in OvCa and normal ovarian tissue samples (*n* = 6); β-actin served as the loading control. **F**, **G** Overall survival (OS) and progression-free survival (PFS) analyses were performed with Kaplan–Meier plotter (http://www.kmplot.com) (Affymetrix ID: 225651_at). **H** Kaplan–Meier survival analysis was conducted by log-rank test (*n* = 88).

Based on the Oncomine, Gene Expression Profiling Interactive Analysis (GEPIA) and The Human Protein Atlas online databases, we first confirmed that the mRNA and protein expression levels of UBE2E2 were elevated in OvCa specimens compared with normal ovary samples, but no significant difference in the DNA level was found (Fig. 1A, B). We performed IHC staining and western blot analysis using 88 OvCa and 26 normal ovarian tissue samples and found that UBE2E2 protein was highly expressed in OvCa tissues but weakly expressed in normal tissues (Fig. 1C–E). Additionally, the results of overall survival (OS) and progression-free survival (PFS) analyses showed that patients with high levels of UBE2E2 experienced significantly worse clinical outcomes. Survival information was obtained from online KM-Plotter database (Fig. 1F, G) and patients enrolled in our study (Fig. 1H and Table S3). Thus, we concluded that UBE2E2 might promote the progression of OvCa and is expected to be a promising indicator of patient prognosis.

### Silencing UBE2E2 expression suppresses the proliferation and migration of OvCa cells

**Fig. 2:**
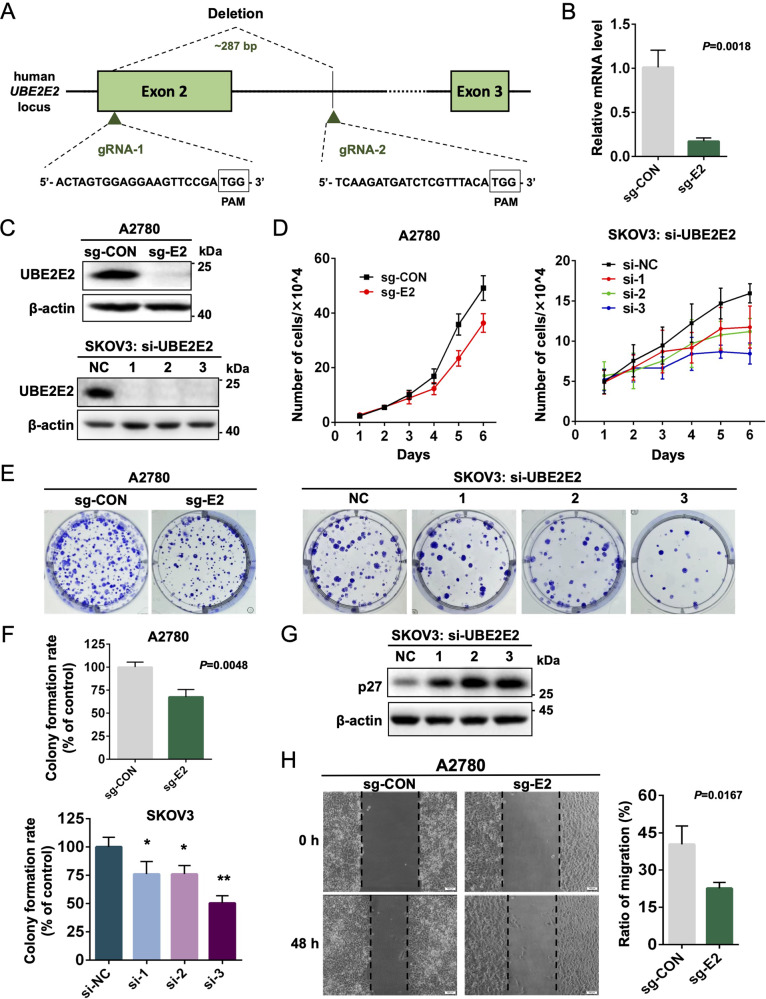
Silencing UBE2E2 expression suppresses the proliferation and migration of OvCa cells. **A** Schematic diagram showing guide RNAs (gRNAs) targeting the human *UBE2E2* exon 2 locus. The protospacer adjacent motif (PAM) sequences are framed in black boxes. **B** qRT–PCR analysis and **C** western blot analyses were performed in A2780 and SKOV3 cells to verify the efficiency of UBE2E2 silencing (sg-CON: sgRNA-control; sg-E2: sgRNA-UBE2E2). **D** Cell counting assays and **E**, **F** colony formation assays were performed to investigate the effect of UBE2E2 silencing on the proliferation of A2780 and SKOV3 cells (*n* = 3). **G** The protein expression level of p27 in SKOV3 cells was assessed by immunoblotting 72 h after siRNA transfection. **H** The migration of A2780 cells was determined by wound healing assay, as described in the “Materials and methods” (*n* = 3). (*p < 0.05, **p < 0.01).

Because UBE2E2 is overexpressed in OvCa, we knocked out UBE2E2 expression in A2780 cells using the CRISPR–Cas9 system (Fig. 2A) and knocked down UBE2E2 expression in SKOV3 cells with siRNA transfection. The silencing efficiency was verified by qRT–PCR or western blotting (Fig. 2B, C). After silencing UBE2E2, the proliferation rate (Fig. 2D) and colony formation rate (Fig. 2E, F) of SKOV3 and A2780 cells were significantly reduced. The knockdown of UBE2E2 in SKOV3 cells enhanced the expression of the cyclin-dependent kinase (CDK) inhibitor p27 (Fig. 2G). In addition, UBE2E2 deletion decreased the migratory ability of A2780 cells compared with the effect in controls (Fig. 2H). These results suggested that the silencing of UBE2E2 can significantly inhibit the proliferation and migration of OvCa cells.

### UBE2E2 activates the Snail signaling pathway to induce EMT in OvCa cells

**Fig. 3:**
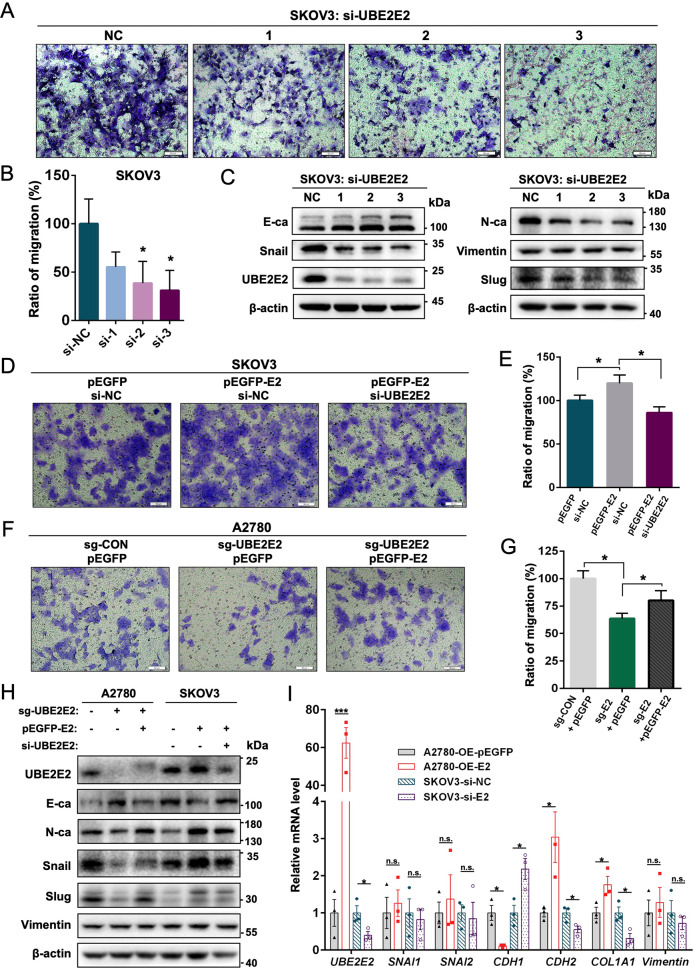
UBE2E2 induces EMT in OvCa cells. **A** Representative images of SKOV3 cell migration after transfection with negative control (NC) siRNA or UBE2E2 siRNA (scale bar = 50 μm). **B** The migration ratio was calculated by dividing the average number of migrated cells in the UBE2E2 siRNA group by the average number of migrated cells in the NC group (*p < 0.05 versus NC). **C** Western blot analysis of EMT markers in SKOV3 cells was performed 72 h after siRNA transfection. **D** Representative images of SKOV3 cell migration. SKOV3 cells were transfected with NC or UBE2E2 siRNA 24 h after transfection with pEGFP-C1 or pEGFP-C1-UBE2E2 plasmids (scale bar = 50 μm). **E** The migration ratio was calculated by dividing the average number of migrated cells in the treated group compared with the control group. **F** Representative images of A2780 cell migration after transfection with pEGFP-C1 or pEGFP-C1-UBE2E2 plasmids (scale bar = 50 μm). **G** The migration ratio was calculated by dividing the average number of migrated cells in the treated group compared with the control group. **H** Western blot analysis of EMT markers in OvCa cells. **I** qRT–PCR analysis of UBE2E2 and EMT markers in OvCa cells (*n* = 3, *p < 0.05, ***p < 0.005). n.s. no significant differences detected, OE overexpression, E2 UBE2E2.

We then explored the role of UBE2E2 in cell migration by performing Transwell assays. As shown in Fig. 3A, B, a significant decrease in migratory capability was found in SKOV3 cells transfected with UBE2E2 siRNA compared with cells transfected with control siRNA. Because EMT is an important cellular program in tumor migration, we thus verified the role played by UBE2E2 in EMT induction. UBE2E2 silencing downregulated the protein expression of Snail, Slug, and a mesenchymal marker (N-cadherin) and increased the expression of an epithelial marker (E-cadherin), whereas the expression of vimentin was not significantly affected (Fig. 3C). Moreover, UBE2E2 overexpression enhanced the migration ability of SKOV3 cells and this effect could be reversed by further knockdown of UBE2E2 (Fig. 3D, E). The migratory ability of A2780 cells was significantly suppressed after UBE2E2 deletion, and re-expression of UBE2E2 could partially rescued the phenotype (Fig. 3F, G). Similarly, the suppression of EMT markers caused by UBE2E2 deletion was reversed by UBE2E2 overexpression, and UBE2E2 silencing partially diminished the promoting effect of UBE2E2 in EMT (Fig. 3H). In addition, the mRNA expression levels of *E-cadherin/CDH1*, *N-cadherin/CDH2* and collagen type I alpha 1 chain (*COL1A1*), target genes of Snail, were significantly influenced by UBE2E2 (Fig. 3I). However, the regulation of UBE2E2 expression exerted no significant effect on the mRNA expression levels of *SNAI1*, *SNAI2/Slug*, and *Vimentin* (Fig. 3I). These data suggest that UBE2E2 plays an important role in OvCa cell migration and EMT induction.

### UBE2E2 enhances Snail stability by inhibiting its ubiquitination

**Fig. 4:**
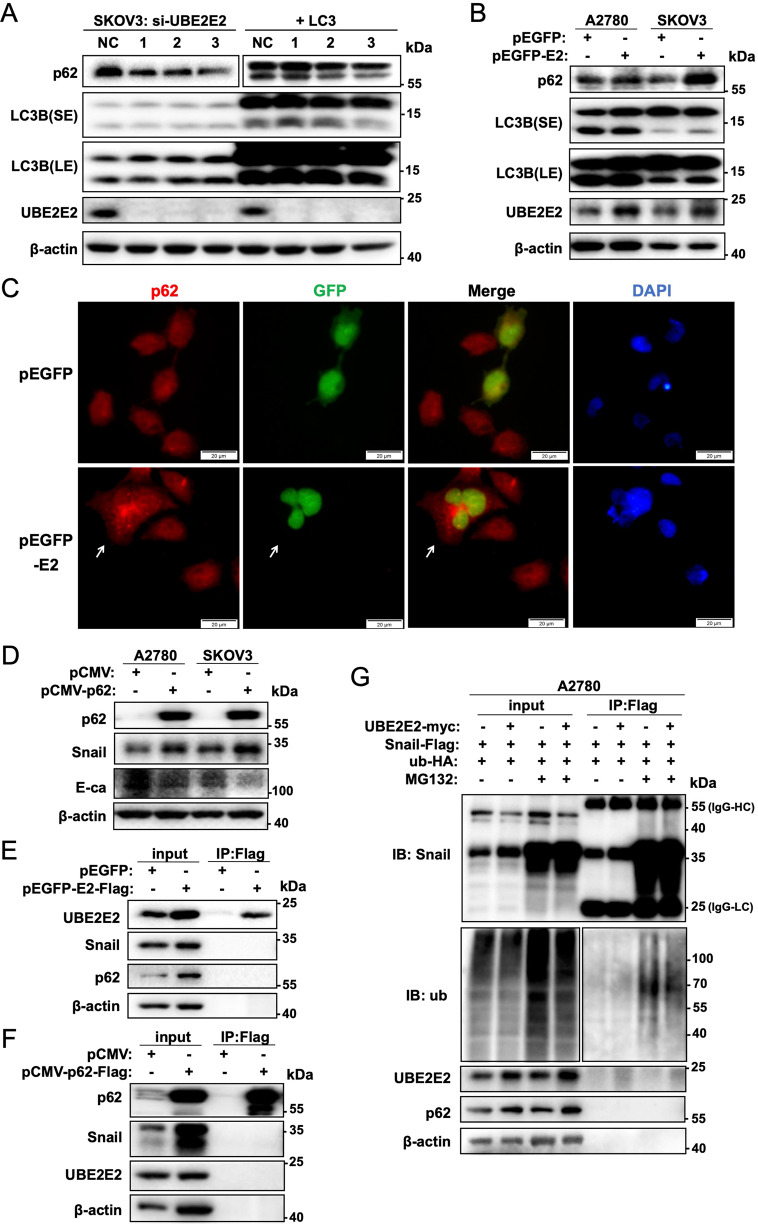
UBE2E2 enhances p62 expression and Snail stability. **A**, **B** The expression of p62, LC3B and UBE2E2 was explored by western blot analysis performed 72 h after cell transfection with UBE2E2 siRNA or overexpression vector. SKOV3 cells were infected with adenoviral particles that directed the expression of mRFP-GFP-LC3, incubated for 24 h and subjected to western blot analysis (SE, short exposure, and LE, long exposure). **C** The changes in p62 expression were assessed by SKOV3 cell immunofluorescence staining. An overlay of red (p62) and green (GFP) channels is shown. White arrows indicate the transfected cells (scale bar = 20 μm). **D** The expression of EMT markers was explored by western blotting performed 72 h after transfection with the p62-expressing vector or control vector. **E**, **F** Immunoprecipitation (IP) of Flag-tagged UBE2E2 and p62 in A2780 cells. **G** Immunoprecipitation (IP) assays were performed to detect the ubiquitination of Snail in A2780 cells 72 h after transfection with the plasmids as shown. MG132, which is used to block the proteasome system, was added to the cultures (final concentration of 10 μM) 12 h prior to cell harvesting. The IgG heavy chain (55-kDa band) and light chain (25-kDa band) were used as markers.

Our experiments showed that UBE2E2 regulates Snail expression at the protein level rather than the mRNA level, which suggests that it is regulated post-translationally. P62 was recently implicated in ubiquitination and proteasome degradation of the Snail protein. As shown in Fig. 4A, the knockdown of UBE2E2 in SKOV3 cells reduced the expression of p62. qRT–PCR (Fig. S2A) and western blot analysis (Fig. 4B) showed that UBE2E2 upregulated the expression of p62 at both the mRNA and protein levels. Immunofluorescence assays revealed that UBE2E2, which is localized in the nucleus, promoted the expression of p62 in the cytoplasm (Fig. 4C). However, UBE2E2 overexpression or silencing did not significantly affect LC3B expression (Fig. 4A, B) or autophagosome formation (detected as GFP-RFP-LC3 puncta) (Fig. S2B).

We then verified the role of p62 in regulating the expression of Snail in OvCa cells. As shown in Fig. 4D, p62 overexpression increased the protein level of Snail and the repression of E-cadherin expression in SKOV3 and A2780 cells. To investigate the interaction between these factors, Flag-tagged UBE2E2 and p62 vectors were transfected into cells, and co-IP assays were performed. We did not observe any direct interactions between UBE2E2 and p62 or Snail (Fig. 4E), or physical interaction between p62 and Snail protein (Fig. 4F). Next, A2780 cells were simultaneously transfected with Flag-tagged Snail, HA-tagged ubiquitin and myc-tagged UBE2E2 plasmids and the ubiquitination of Snail was detected by immunoprecipitation. The results revealed that UBE2E2 overexpression reduced the ubiquitination of Snail protein (Fig. 4G). Furthermore, overexpression of UBE2E2 or p62 or Nrf2 in UBE2E2-depleted cells could partially rescue the Snail-induced EMT (Fig. S2C). These findings indicate that UBE2E2 induces EMT in OvCa cells by upregulating p62 expression, which leads to reduced ubiquitination and increased stability of Snail.

### UBE2E2 modulates Nrf2 expression and nuclear translocation

**Fig. 5:**
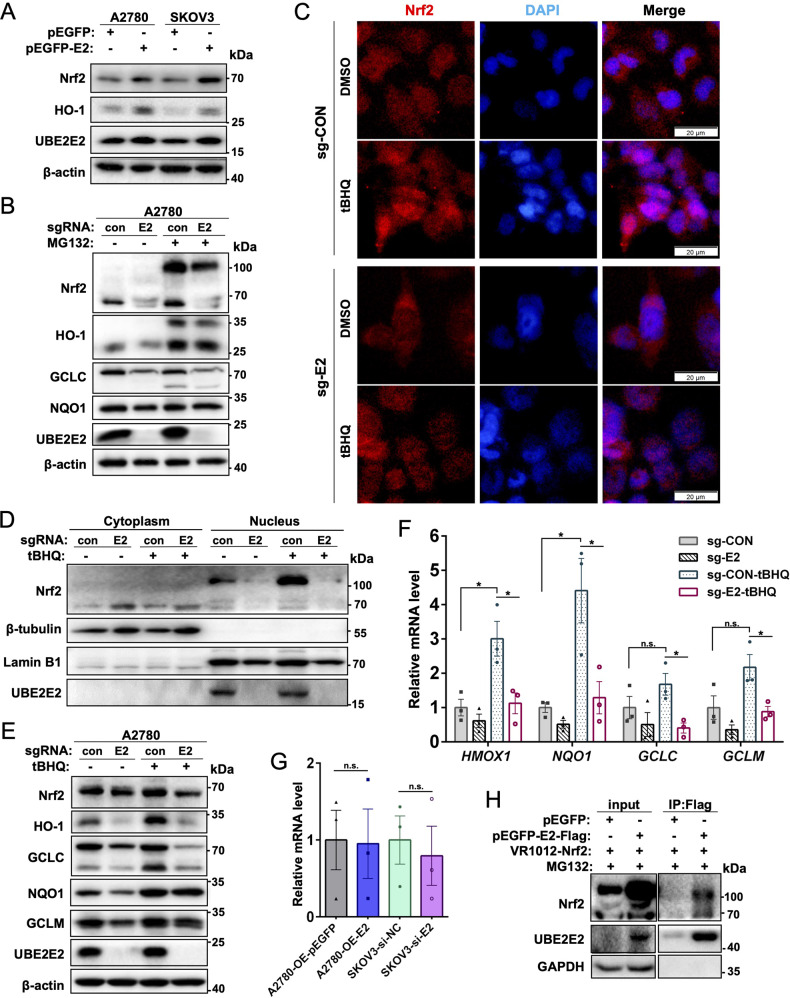
UBE2E2 regulates Nrf2 expression and nuclear translocation. **A**, **B** The expression of Nrf2 pathway proteins was explored by western blotting with β-actin as the loading control. MG132, which is used to block the proteasome system, was added to the cultures (final concentration of 10 μM) 12 h prior to cell harvesting. **C** The subcellular distribution of Nrf2 was assessed by immunofluorescence staining. An overlay of blue (nuclei) and red (Nrf2) channels is shown (scale bar = 20 μm). **D** The cytoplasm and nuclear fractions were analyzed by immunoblotting with β-tubulin, and Lamin B1 as markers for cytosol and nuclear fractions, respectively. **E** The expression of Nrf2 pathway proteins was detected by immunoblotting. **F** The mRNA levels were measured by qRT–PCR (*p < 0.05; n.s. no significant differences detected). **G** The mRNA expression level of Nrf2 in OvCa cells was measured by qRT–PCR. **H** Immunoprecipitation (IP) of Flag-tagged UBE2E2 in SKOV3 cells.

Typically, p62 inhibits Nrf2 degradation by disrupting the Keap1-Nrf2 interaction, which leads to Nrf2 stabilization and nuclear translocation [17]. We then tested whether UBE2E2 affects the nuclear accumulation and transcriptional activity of Nrf2 in homeostatic or oxidatively stressed cells. As shown in Fig. 5A, B, overexpression of UBE2E2 in A2780 and SKOV3 cells upregulated the expression of Nrf2 and its target genes, whereas inhibition of UBE2E2 expression had the opposite effect. Immunofluorescence staining and western blot analysis of the subcellular fractions were then performed to assess the nuclear localization of Nrf2. Exposure to tBHQ, a potent Nrf2 activator, increased the amount of Nrf2 protein translocated to the nucleus in control cells, but this protective effect was abrogated in UBE2E2-depleted cells (Fig. 5C, D). Furthermore, the western blot (Fig. 5E) and qRT–PCR (Fig. 5F) analyses also showed that tBHQ effectively activated the Nrf2 signaling pathway in A2780 control cells but not in UBE2E2-knockout cells. While UBE2E2 exerted no significant effect on Nrf2 mRNA expression (Fig. 5G), it was found associated with Nrf2 protein in SKOV3 cells by co-IP (Fig. 5H). Taken together, our findings demonstrate that UBE2E2 can enhance the expression and nuclear translocation of Nrf2 and thereby regulate downstream antioxidant enzyme catalase activity.

### The N-terminal of UBE2E2 is required for its interaction with Nrf2

**Fig. 6:**
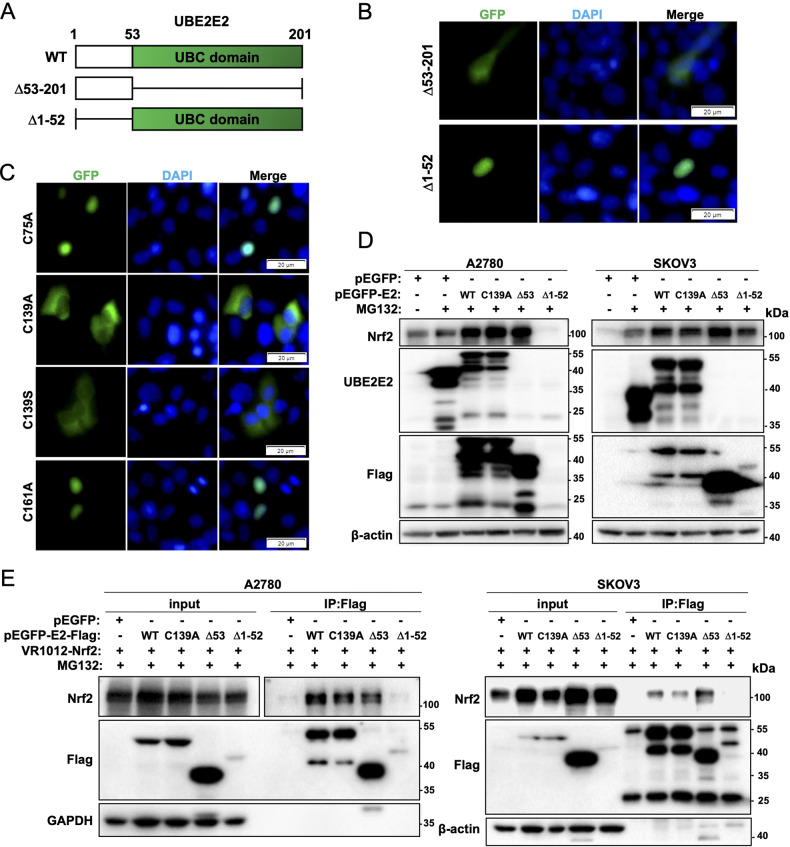
The N-terminal of UBE2E2 is required for its interaction with Nrf2. **A** Schematic diagram showing the full-length and truncated forms of UBE2E2. The numbers indicate the positions of the amino acids. WT wild-type. **B** Effects of the truncation mutation on the UBE2E2 intracellular distribution. An overlay of blue (nuclei) and green (GFP) channels is shown. Scale bar = 20 μm. **C** Effects of mutations on the UBE2E2 intracellular distribution in SKOV3 cells. An overlay of blue (nuclei) and green (GFP) channels is shown (scale bar = 20 μm). **D** The expression of Nrf2, Flag and UBE2E2 was explored by western blot analysis 72 h after transfection with WT or mutant UBE2E2 expression vectors. MG132 was added to the cultures 12 h prior to cell harvesting. β-actin was used as the loading control. Δ53 Δ53-201. **E** Immunoprecipitation (IP) of Flag-tagged WT or mutant UBE2E2 in OvCa cells.

A2780 and SKOV3 cells transfected with GFP-UBE2E2 expression vectors exhibited fusion proteins localized to nuclei (Figs. 4C and S3A). As a class III E2, UBE2E2 comprises a UBC domain and an NH2-terminal extension, and the Ub-loaded form of UBE2E2 selectively interacts with the transport receptor importin-11 [18]. We then generated two UBE2E2 deletion mutants (Fig. 6A) and observed that the UBE2E2 truncation mutant lacking the first 52 N-terminal amino acids (Δ1-52) was predominantly expressed in the nucleus, whereas the C-terminal-truncated UBE2E2 mutant (Δ53-201) was primarily distributed in the cytoplasm (Fig. 6B). We then generated constructs with point mutations at the putative active site cysteine (Cys139) and two other cysteine residues. The active site UBE2E2 mutant bearing either a serine (C139S) or alanine (C139A) substitution displayed reduced nuclear translocation, whereas the C75A and C161A mutants followed the distribution pattern of wild-type (WT) UBE2E2 (Fig. 6C). In addition, compared with WT UBE2E2 or other mutants, the C139S and C139A mutants showed significantly impaired p62 and Snail expression induction (Fig. S3B). Together, these experiments support the notion that the integrity of the active site Cys139 residue is critical for the cellular location of UBE2E2 and influences its functions in EMT induction. Unexpectedly, both C139A mutant and the N-terminal of UBE2E2 (C-terminal-truncated UBE2E2 mutant Δ53-201) were found to cause Nrf2 accumulation, which was similar to that of WT UBE2E2 (Fig. 6D). The undegraded proteasome substrates (Nrf2 in 110 kDa) and polymerized forms of UBE2E2 enzyme would be detectable in the presence of proteasome inhibitor, MG132. We further confirmed that both C139A mutant and the N-terminal of UBE2E2 could interact with Nrf2 in A2780 and SKOV3 cells (Fig. 6E). Meanwhile, immunofluorescence staining indicated co-localization of C139A-UBE2E2 and Nrf2 in A2780 cells (white arrows) co-transfected with GFP-C139A-UBE2E2 and Nrf2 expression vectors (Fig. S3C). And over-expression of C139A-UBE2E2 in UBE2E2-depleted cells mainly caused cytoplasmic Nrf2 accumulation (Fig. S3D). Hence, the aforementioned results unraveled that the N-terminal of UBE2E2 was required for its interaction with Nrf2, but its function in Nrf2 nuclear translocation might still rely on its Cys139 residue.

### Loss of UBE2E2 impairs OvCa cell invasion and proliferation in vivo

**Fig. 7:**
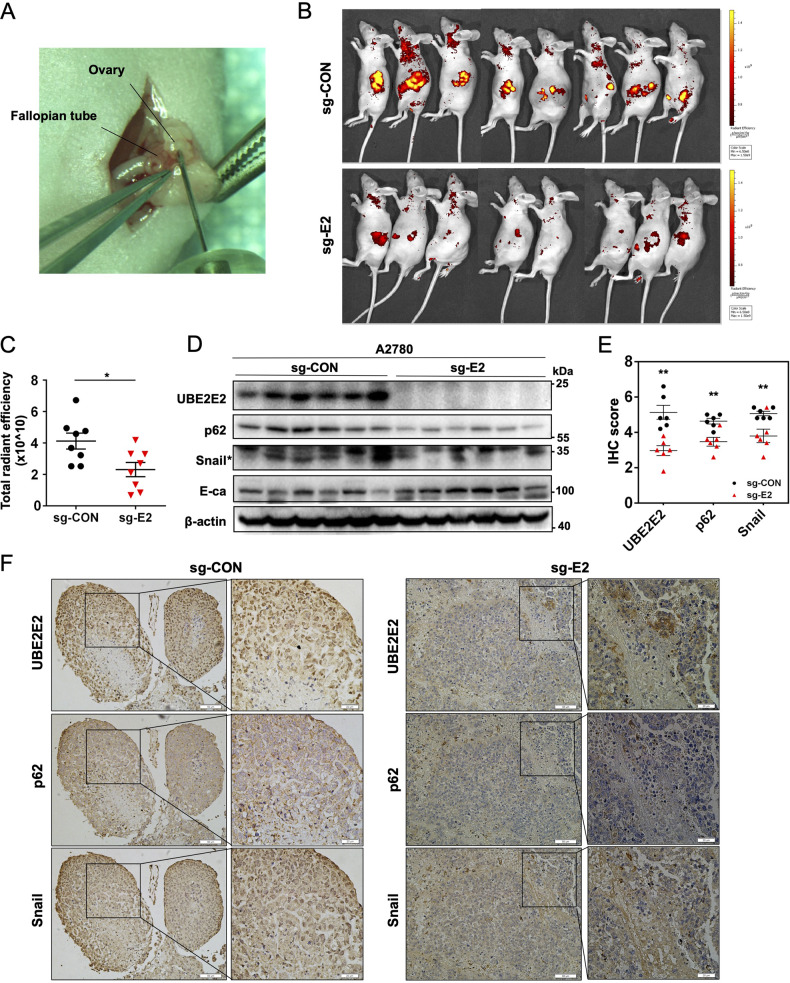
Loss of UBE2E2 expression inhibits OvCa cell invasion and proliferation in vivo. **A** Illustration of orthotopic implantation of A2780 cells into ovarian bursa. **B** Fluorescence imaging of the tumor site (radiant efficiency, [p/s/cm^2^/sr]/[μW/cm^2^]) (*n* = 8). **C** For fluorescence quantification, regions of interest (ROIs) were drawn with Living Imaging 3.0 software, and the total radiant efficiency was determined (*p < 0.05). The expression of the p62, Snail, and E-cadherin proteins in xenograft tumors was explored by (**D**) western blot and (**E**) immunohistochemical (IHC) analyses (*n* = 6, **p < 0.01). The asterisk indicates the protein band of Snail. **F** Representative IHC images of tumor samples collected from sacrificed mice (×20 magnification, scale bar = 50 μm; ×40 magnification, scale bar = 20 μm).

To determine whether UBE2E2 downregulation would affect the invasion and proliferation of OvCa cells in vivo, control cells or UBE2E2-knockout cells were transplanted orthotopically (Fig. 7A) or injected intraperitoneally into BALB/c nude mice. Fluorescence imaging and average radiance values indicated that UBE2E2-depleted OvCa cells formed fewer tumor nodules than the control cells in orthotopic OvCa xenograft models (Fig. 7B, C) and intraperitoneal xenograft models (Fig. S4A, B). No significant difference in average body weight was found between the UBE2E2-depleted group and the control group (Fig. S4C). Furthermore, western blot and IHC analyses showed that UBE2E2 deletion decreased the levels of p62 and Snail and increased the E-cadherin level in xenograft tumors (Fig. 7D–F). Thus, a lack of UBE2E2 impairs the ability of OvCa cells to grow into tumors in vivo.

## Discussion

Over the past decade, emerging evidence has revealed the roles played by certain Ub-conjugating enzymes (E2s) in promoting tumorigenesis and chemotherapy resistance in malignancies, including OvCa [19, 20]. Because E2s are considered novel potential drug targets, several specific inhibitors of E2 proteins have been identified and developed [21, 22]. Therefore, we were interested in investigating the tumorigenic mechanisms of E2s closely related to the prognosis of OvCa patients. By performing bioinformatics analyses, we found that UBE2E2 was upregulated and associated with poor prognosis in OvCa. Thus far, UBE2E2 has been shown to interact with several known E3 ligases, including ARA54, RNF8, BRCA1-BARD1, CHIP, NleG2-3 effector, Dorfin, and Mulan [23, 24]. In the present study, we clarified the underlying molecular mechanisms of UBE2E2 in OvCa progression.

Posttranslational modifications such as ubiquitination, phosphorylation, and lysine oxidation collectively influence Snail protein stability, cellular localization, and activity [5]. P62, an autophagic receptor, binds to the autophagic membrane protein Atg8/LC3 and recruits target proteins to autophagosomes [25]. Snail ubiquitination partially depends on its phosphorylation by GSK-3β, and p62 reportedly serves as a shuttle factor to promote the interaction between GSK-3β and the proteasome and ultimately activate the Snail signaling pathway [7]. Additionally, Zada et al. found that Snail proteins are physically associated and colocalized with p62 and LC3 in cancer cells [26]. The results of other studies addressing the Snail-p62 interaction under certain conditions (hypoxia, high glucose, and TGFβ treatment) [26, 27] suggest that autophagy controls the Snail levels in synergy with and as an alternative to the UPS. Collectively, these data indicate that p62 exerts important regulatory effects on Snail degradation. In this study, we showed that the overexpression of p62 increased the Snail levels, but co-IP assays did not reveal direct binding of p62 to Snail. We also failed to detect any effects of UBE2E2 on the expression of LC3B or autophagosome formation. Therefore, we speculated that UBE2E2 might modulate the Snail protein expression levels mainly through proteasome degradation rather than through autophagy-dependent degradation. Our results indicated that the overexpression of UBE2E2 reduced the ubiquitination of Snail, but we did not detect any physical interaction between UBE2E2 and p62 or UBE2E2 and Snail. The present study provides one possible mechanism by which UBE2E2 enhances the Snail-mediated EMT can involve the up-regulation of p62 induced by UBE2E2, and the data do not rule out the possibility of weak or transient interactions between the proteins.

The antioxidant capacity of OvCa plays a crucial role in cell survival and metastasis, and Nrf2 is the major regulator of the endogenous antioxidant defense system [28]. As mentioned above, p62 contributes to the activation of the Nrf2 signaling pathway by creating a positive feedback loop. In addition, Plafker et al. reported that three class III E2 proteins, UbcM2, UbcM3/UBE2E1, and UBE2E2, bind to recombinant H6-S-Nrf2 in the presence of *N*-ethylmaleimide (NEM) [29]. Here, we demonstrated that the overexpression of UBE2E2 upregulated the expression levels of Nrf2 and its target genes, whereas the Nrf2 activator tBHQ failed to stimulate the Nrf2 pathway in UBE2E2-depleted cells. Moreover, UBE2E2-deficient OvCa cells exhibited impairment of forming clones in vitro and growing into tumors in vivo, which may have been partially due to a lowered antioxidant capacity.

Previous studies have reported that the abovementioned three class III E2 proteins (UbcM2, UbcH6/UBE2E1 and UBE2E2) are imported into the nucleus by the transport receptor importin-11, which requires a covalent connection between Ub and the active site cysteine. Considering these findings, we confirmed that the UBC domain (residues 53-201) of UBE2E2 is sufficient to induce protein translocation into the nucleus. Typically, the central active-site cysteine (Cys), which binds ubiquitin through a thioester bond, is approximately located at the 87th amino acid position in the UBC domain. Our study showed that C139A mutant can reduce the ability of UBE2E2 to accumulate in the nucleus and induce p62 or Snail expression. Moreover, the N-terminal of UBE2E2 (residues 1-52) is necessary and sufficient to interact with Nrf2. The co-localization and accumulation of C139A-UBE2E2 and Nrf2 in the cytoplasm of OvCa cells indicated that the function played by UBE2E2 in Nrf2 nuclear translocation might still rely on its Cys139 residue. It’s also been reported that UBE2E3 may promote Nrf2 transcriptional activity by restricting Nrf2 partitioning to mitochondria and inhibiting the repressive activity of nuclear Keap1, the major suppressor of Nrf2 [30]. These observations suggest that the mechanisms through which UBE2E2 regulates the localization and activity of Nrf2 may be even more complicated. A single amino acid, Cys139, is responsible for the cellular distribution and function of UBE2E2, which may increase the possibility of structure-based inhibitor development.

**Fig. 8:**
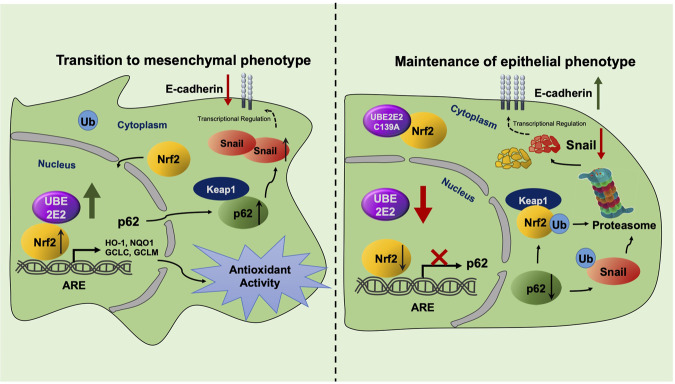
Model illustrating the role played by UBE2E2 in OvCa cell progression. UBE2E2 enhances epithelial-mesenchymal transition and antioxidant capacity of OvCa cells via modulation of Nrf2/p62 and Snail signaling.

In conclusion, our study provides new insights into the role of UBE2E2 in OvCa progression, metastasis and prognosis (Fig. 8). Our results suggest that UBE2E2 enhances the antioxidant capacity and EMT of OvCa cells by upregulating the expression of p62, promoting the Nrf2-ARE transcriptional regulation, stabilizing the expression of Snail and inhibiting its ubiquitin-mediated degradation. Overall, our data reveal a novel mechanism for UBE2E2 in regulating EMT and metastasis in OvCa, and targeting UBE2E2 may be a promising strategy for treating aggressive OvCa in the future.

## Supplementary information

Supplemental Material

Reproducibility checklist

original data files

## Data Availability

All data generated or analyzed during this study are included in this published article (and its Supplementary Information files).
