# Supplementary material for: UBE2E2 enhances Snail-mediated epithelial-mesenchymal transition and Nrf2-mediated antioxidant activity in ovarian cancer
Source: Cell Death Dis. 2023 Feb 10;14(2):100. doi: 10.1038/s41419-023-05636-z (PMC9918489; doi:10.1038/s41419-023-05636-z)
Supplement: Supplementary file 1 — Supplemental Material [file 41419_2023_5636_MOESM1_ESM.pdf]

Supplementary Figures

Title: UBE2E2 enhances Snail-mediated epithelial-mesenchymal transition and Nrf2-mediated antioxidant activity in ovarian cancer

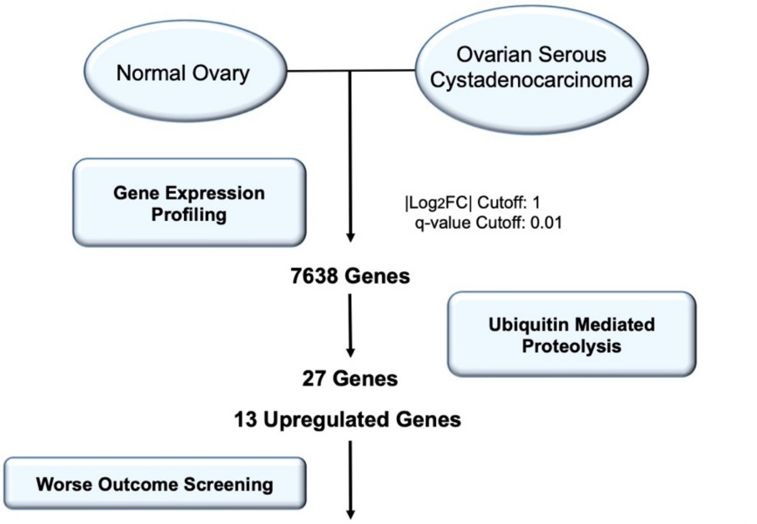

Gene Symbol	Gene Name	Functions Verified Experimentally
UBE2C	ubiquitin conjugating enzyme E2 C	cancer-promoting
UBE2E2	ubiquitin conjugating enzyme E2 E2	cancer-promoting
UBE2F	ubiquitin conjugating enzyme E2 F	cancer-promoting
UBE2K	ubiquitin conjugating enzyme E2 K	cancer-promoting
UBE2N	ubiquitin conjugating enzyme E2 N	cancer-promoting
UBE2S	ubiquitin conjugating enzyme E2 S	cancer-promoting
FBXO2	F-box protein 2	cancer-promoting

Figure S1. Identification of genes associated with detrimental outcomes in OvCa. Transcriptomic expression analyses were performed using the OvCa dataset from the GEPIA database and differentially expressed genes (DEGs) identified with the following criteria: fold change value > 2 and q-value < 0.01. Biological pathway analyses were performed using Kyoto Encyclopedia of Genes and Genomes (KEGG; https://www.genome.jp/kegg/), and outcome screening for overall survival (OS) was carried out using the online Kaplan–Meier (KM) Plotter tool.

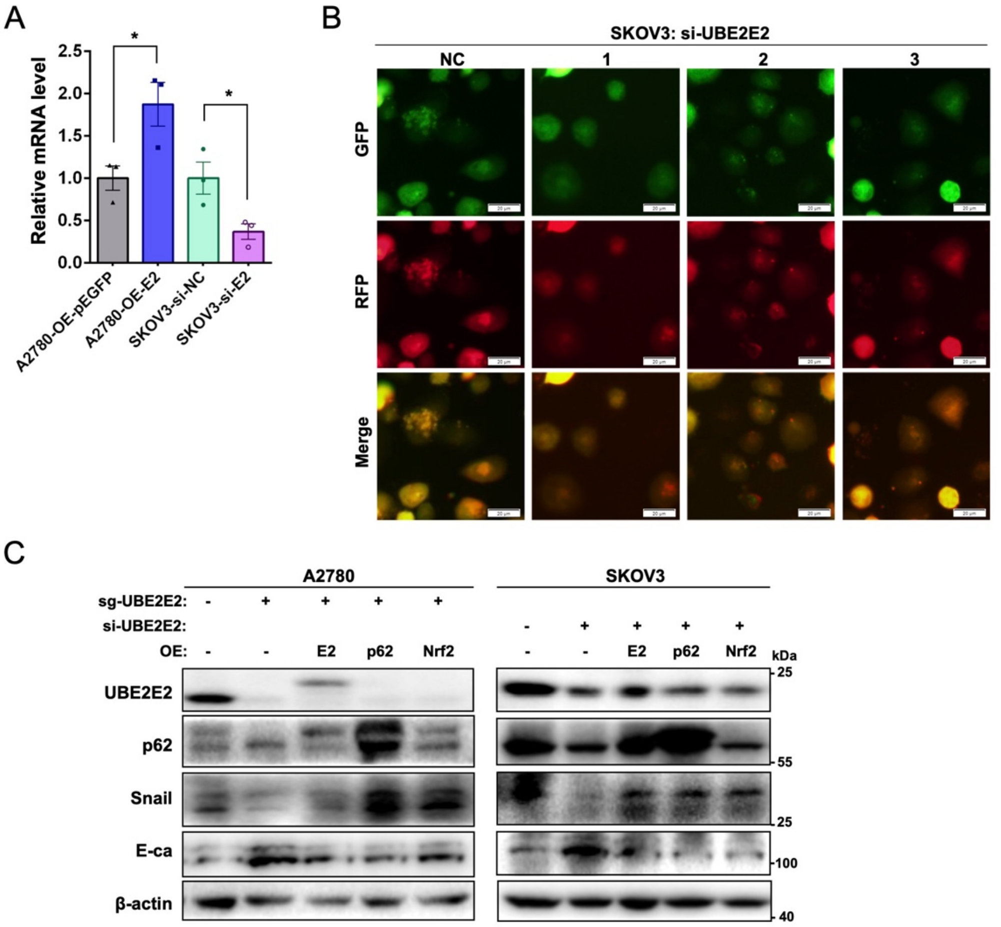

Figure S2. UBE2E2 increases the mRNA level of p62 but exerts no significant effect on autophagy.

(A) The mRNA expression level of p62 in OvCa cells was measured by qRT–PCR (**P* < 0.05). (B) Representative images showing GFP-RFP-LC3 puncta (autophagosomes) and RFP-LC3 puncta (autophagolysosomes) in SKOV3 cells infected with the adenovirus vector containing the mRFP-GFP-LC3 reporter. Scale bar = 20 μm. (C) The expression of the p62, Snail, UBE2E2 and E-cadherin proteins was explored by western blot. Indicated overexpression (OE) plasmids were transfected into A2780 control or UBE2E2-depleted cells. SKOV3 cells were transfected with indicated overexpression plasmids 24 h after transfection with NC (negative control) or UBE2E2 siRNA.

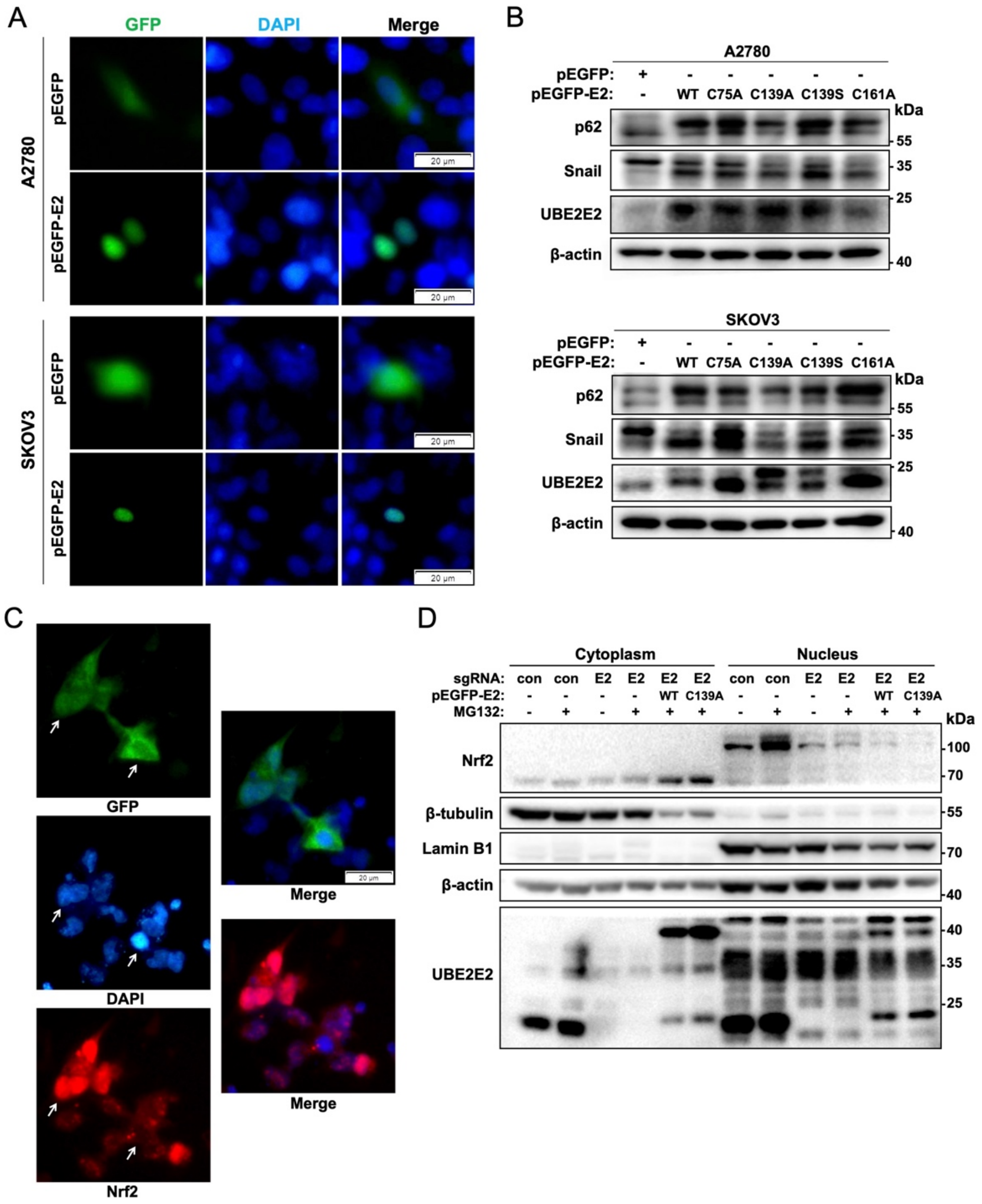

Figure S3. The Cys139 residue is important for UBE2E2 cellular localization and function.

(A) OvCa cells were transfected with pEGFP-C1 or pEGFP-C1-UBE2E2 plasmids, and images of UBE2E2 intracellular distribution were captured using an epifluorescence microscope. An overlay of blue (nuclei) and green (GFP) channels is shown. Scale bar = 20 μm. (B) The expression of p62, Snail and UBE2E2 was explored by western blot analysis 72 h after transfection with WT or mutant UBE2E2 expression vectors. β-actin was used as the loading control. (C) The subcellular distribution of Nrf2 was assessed by immunofluorescence staining. Overlays of blue (nuclei) and green (GFP) channels, blue (nuclei) and red (Nrf2) channels are shown (scale bar = 20 μm). (D) The cytoplasm and nuclear fractions were analyzed by immunoblotting with β-tubulin, and Lamin B1 as markers for cytosol and nuclear fractions, respectively.

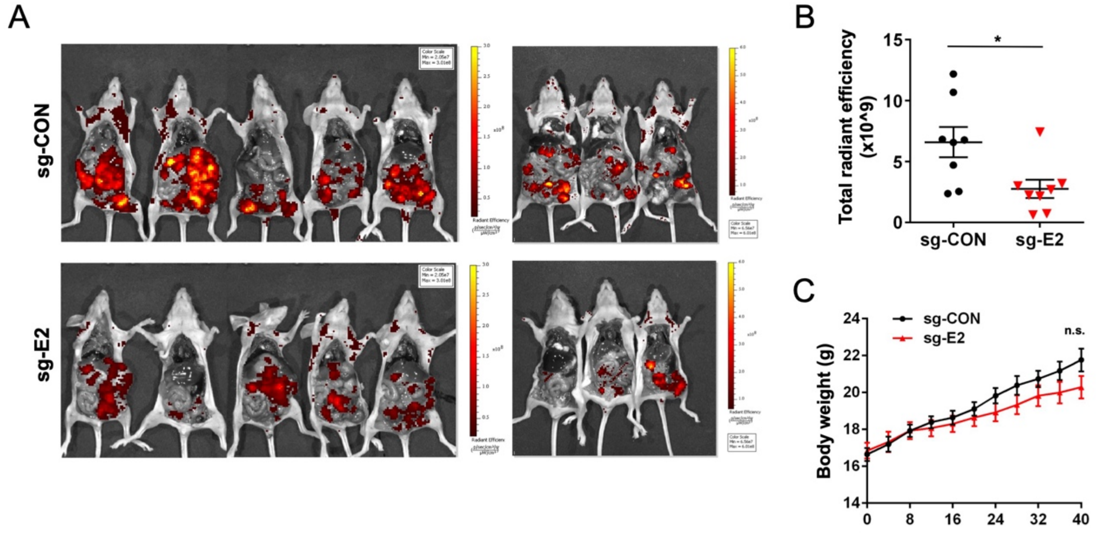

Figure S4. Loss of UBE2E2 expression inhibits OvCa cell invasion and proliferation in vivo.

(A) Fluorescence imaging of the tumor site (radiant efficiency, [p/sec/cm^2/sr]/[μW/cm^2]) (n = 8). (B) For fluorescence quantification, regions of interest (ROIs) were drawn with Living Imaging 3.0 software, and the total radiant efficiency was determined (*P < 0.05). (C) The body weight of the mice was recorded every 4 days. (n.s., no significant differences detected)

Table S1: Sequences of oligonucleotides for siRNA assays.

siRNA	Sequences
si-NC (Negative Control)	Forward: UUCUCCGAACGUGUCACGUTT<br>Reverse: ACGUGACACGUUCGGAGAATT
si-UBE2E2-1	Forward: CCGAUGGAGAUCAACGUGATT<br>Reverse: UCACGUUGAUCUCCAUCGGTT
si-UBE2E2-2	Forward: CGUGAAAGUGUUCAGCAAGTT<br>Reverse: CUUGCUGAACACUUUCACGTT
si-UBE2E2-3	Forward: GACCCAAAGGAGACAACAUTT<br>Reverse: AUGUUGUCUCCUUUGGGUCTT

Table S2: Primer sequences used for qRT-PCR assays.

Gene	Sequences
UBE2E2	Forward: 5’-GTGAAAGTGTTCAGCAAGAACCA-3’<br>Reverse: 5’-GTTGACAATTTAGCAGCGGTTTT-3’
SNAI1	Forward: 5’-TCGGAAGCCTAACTACAGCGA-3’<br>Reverse: 5’-AGATGAGCATTGGCAGCGAG-3’
SNAI2	Forward: 5’-CGAACTGGACACACATACAGTG-3’<br>Reverse: 5’-CTGAGGATCTCTGGTTGTGGT-3’
CDH1	Forward: 5’-AGTGGGCACAGATGGTGTGA-3’<br>Reverse: 5’-TAGGTGGAGTCCCAGGCGTA-3’
COL1A1	Forward: 5’-GAGGGCCAAGACGAAGACATC-3’<br>Reverse: 5’-CAGATCACGTCATCGCACAAC-3’
VIMENTIN	Forward: 5’-AGTCCACTGAGTACCGGAGAC-3’<br>Reverse: 5’-CATTTCACGCATCTGGCGTTC-3’
HMOX1	Forward: 5’-ACTGCGTTCCTGCTCAACATCCA-3’<br>Reverse: 5’-TGGGAGTCTCCACGGGGGCAGAAT-3’
NQO1	Forward: 5’-CTGGAAGCCGCAGACCTTGTG-3’<br>Reverse: 5’-CCTTGCAGAGAGTACATGGAG-3’
GCLC	Forward: 5’-TCAGGCTCTTTGCACAATAAC-3’<br>Reverse: 5’-ATTGGTACATTGATGACAACC-3’
GCLM	Forward: 5’-GAATGGAGTTCCCAAATCAAC-3’<br>Reverse: 5’-GCAACTCCAAGGACTGAACAG-3’
p62	Forward: 5’-AGGCGCACTACCGCGAT-3’<br>Reverse: 5’-CGTCACTGGAAAAGGCAACC-3’
CDH2	Forward: 5’-CCACGCCGAGCCCCAGTATC-3’<br>Reverse: 5’-CCCCCAGTCGTTCAGGTAATCA-3’
NFE2L2	Forward: 5’-CCAATTCAGCCAGCCCAGCACAT-3’<br>Reverse: 5’-CAGGTGACTGAGCCTGATTAGTAG-3’

Table S3: Clinicopathological characteristics of tumor samples.

Patient Number	Age (Years)	Side and Tumor Location	TNM stage	IHC score of UBE2E2	UBE2E2 Expression Level	Survival Status	Survival (Months)
1	49	bilateral ovaries	IIA	2.2	Low	Alive	over 93
2	52	bilateral ovaries	IIIB	1.8	Low	Alive	over 81
3	41	right ovary	IA	2.6	Low	Dead	37
4	53	left ovary	IC	5.4	High	Alive	over 73
5	58	left ovary	IIB	3	Low	Dead	28
6	35	left ovary	IIA	1.8	Low	Alive	over 71
7	48	bilateral ovaries	IC	2	Low	Alive	over 70
8	52	bilateral ovaries	IIA	2.2	Low	Alive	over 69
9	53	bilateral ovaries	IB	5.2	High	Dead	28
10	44	right ovary	IA	2.2	Low	Alive	over 76
11	42	right ovary	IIIC	2.4	Low	Alive	over 63
12	49	bilateral ovaries	IV	4.8	High	Alive	over 63
13	64	right ovary	IC	3.8	High	Alive	over 60
14	56	bilateral ovaries	IIIA	2.8	Low	Alive	over 57
15	32	right ovary	IC	2.2	Low	Alive	over 55
16	52	bilateral ovaries	IIIC	5.2	High	Dead	31
17	62	bilateral ovaries	IIIC	4.6	High	Dead	1
18	74	bilateral ovaries	IIB	2	Low	Dead	33
19	46	bilateral ovaries	IIIC	5	High	Dead	44
20	74	right ovary	IIIC	4	High	Dead	40
21	52	right ovary	IIIC	2.6	Low	Dead	23
22	53	bilateral ovaries	IIIC	3.6	High	Dead	24
23	55	bilateral ovaries	IIIC	2	Low	Alive	over 47
24	54	bilateral ovaries	IIIC	3	Low	Alive	over 43
25	55	bilateral ovaries	IC	3.4	Low	Alive	over 42
26	65	bilateral ovaries	IIIC	6.4	High	Dead	16
27	58	bilateral ovaries	IIIA	3.4	Low	Dead	9
28	52	bilateral ovaries	IIB	2.8	Low	Alive	over 40
29	63	bilateral ovaries	IIIC	2.8	Low	Alive	over 44
30	21	bilateral ovaries	IC	2.2	Low	Alive	over 42
31	57	bilateral ovaries	IIA	2.4	Low	Alive	over 42
32	62	left ovary	IIA	5.8	High	Dead	17
33	51	bilateral ovaries	IIIC	3.6	High	Alive	over 96
34	44	bilateral ovaries	IIIC	2.6	Low	Alive	over 95
35	48	bilateral ovaries	IIIB	3.2	Low	Dead	43
36	57	bilateral ovaries	IIIC	4.6	High	Dead	16
37	72	bilateral ovaries	IV	4.2	High	Alive	over 85
38	57	bilateral ovaries	IIIB	2.2	Low	Dead	34
39	59	bilateral ovaries	IIIC	6.4	High	Dead	36
40	57	left ovary	IIB	3.8	High	Alive	over 77
41	60	bilateral ovaries	IIIB	6.6	High	Dead	39
42	65	bilateral ovaries	IIIB	6.8	High	Alive	over 77
43	64	bilateral ovaries	IIIC	2.8	Low	Alive	over 76
44	61	bilateral ovaries	IIIB	6.6	High	Alive	over 75
45	53	bilateral ovaries	IIIC	3	Low	Alive	over 73

Patient Number	Age (Years)	Side and Tumor Location	TNM stage	IHC score of UBE2E2	UBE2E2 Expression Level	Survival Status	Survival (Months)
46	49	bilateral ovaries	IIIB	5.8	High	Dead	41
47	50	bilateral ovaries	IIIC	4.4	High	Alive	over 54
48	61	bilateral ovaries	IIIB	5	High	Dead	27
49	50	bilateral ovaries	IB	4.6	High	Alive	over 66
50	51	bilateral ovaries	IIIC	3.8	High	Alive	over 66
51	45	right ovary	IIIC	5.2	High	Dead	19
52	67	bilateral ovaries	IIIB	5.8	High	Alive	over 63
53	68	bilateral ovaries	IC	3	Low	Alive	over 59
54	56	right ovary	IIA	3.4	Low	Alive	over 59
55	39	bilateral ovaries	IIIC	3.2	Low	Alive	over 59
56	59	left ovary	IA	2.8	Low	Alive	over 61
57	54	bilateral ovaries	IIIC	6.8	High	Dead	5
58	49	bilateral ovaries	IV	5.2	High	Alive	over 55
59	69	bilateral ovaries	IV	3.8	High	Alive	over 54
60	53	bilateral ovaries	IIIB	5.2	High	Dead	14
61	54	bilateral ovaries	IIIB	3.8	High	Alive	over 55
62	52	right ovary	IIIC	5	High	Dead	20
63	66	right ovary	IC	3.4	Low	Alive	over 45
64	48	right ovary	IIB	4.6	High	Alive	over 42
65	48	right ovary	IA	3.2	Low	Alive	over 40
66	52	bilateral ovaries	IIB	3.8	High	Alive	over 40
67	50	right ovary	IIIB	4	High	Alive	over 40
68	75	bilateral ovaries	IIIC	4.6	High	Dead	19
69	69	left ovary	IIIB	3	Low	Alive	over 49
70	47	bilateral ovaries	IIIC	6	High	Alive	over 57
71	55	bilateral ovaries	IV	6.2	High	Alive	over 47
72	48	bilateral ovaries	IIIC	5.6	High	Dead	20
73	48	right ovary	IIIC	4.6	High	Alive	over 42
74	48	bilateral ovaries	IV	3.4	Low	Alive	over 41
75	69	bilateral ovaries	IIIC	4.8	High	Alive	over 40
76	59	bilateral ovaries	IIIC	6.4	High	Dead	32
77	44	bilateral ovaries	IV	6.2	High	Alive	over 42
78	59	left ovary	IIIC	5.8	High	Alive	over 41
79	52	bilateral ovaries	IIIC	6.6	High	Dead	18
80	70	bilateral ovaries	IIIC	6.6	High	Dead	7
81	55	bilateral ovaries	IIIC	5	High	Alive	over 30
82	53	right ovary	IIIC	5.6	High	Alive	over 28
83	52	bilateral ovaries	IIIC	5.4	High	Alive	over 30
84	54	left ovary	IIIA	5.2	High	Alive	over 34
85	46	bilateral ovaries	IIIC	5.4	High	Alive	over 32
86	68	bilateral ovaries	IIIC	5	High	Dead	12
87	31	bilateral ovaries	IIIC	2.6	Low	Alive	over 21
88	69	bilateral ovaries	IIIB	5.4	High	Alive	over 36
