# Supplementary material for: UBE2E2 enhances Snail-mediated epithelial-mesenchymal transition and Nrf2-mediated antioxidant activity in ovarian cancer
Source: Cell Death Dis. 2023 Feb 10;14(2):100. doi: 10.1038/s41419-023-05636-z (PMC9918489; doi:10.1038/s41419-023-05636-z)

Original Images for Blots

Fig.1E

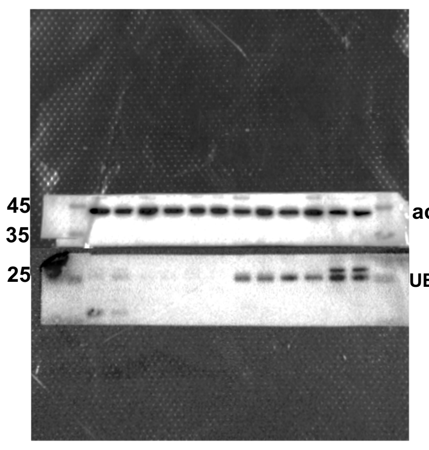

Fig.2C

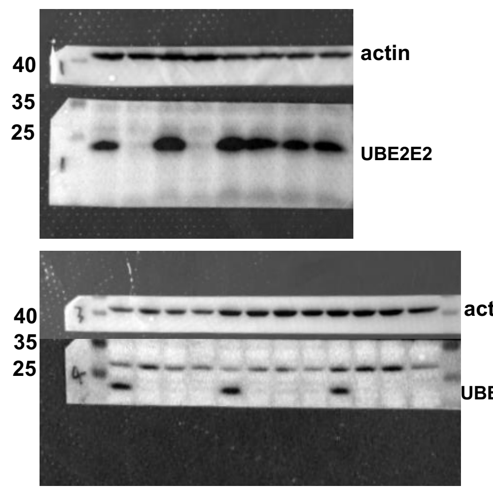

Fig.2G

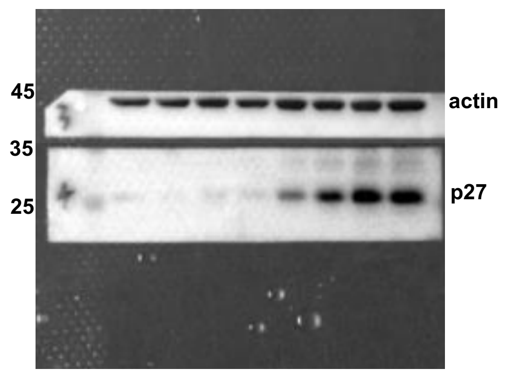

Fig.3C

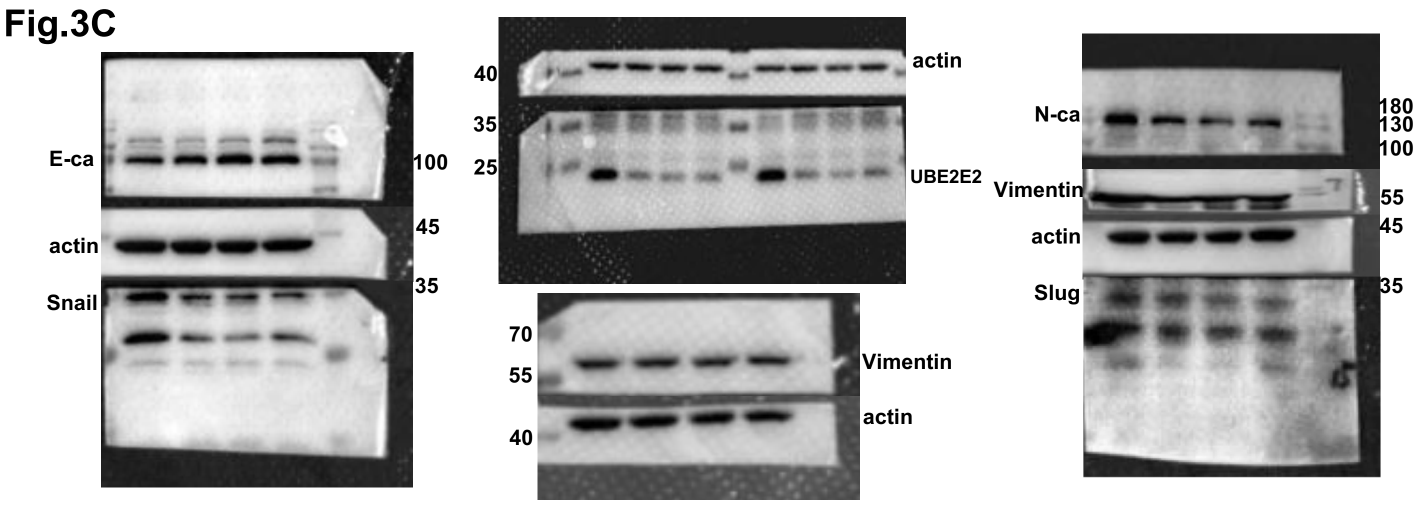

Fig.3H

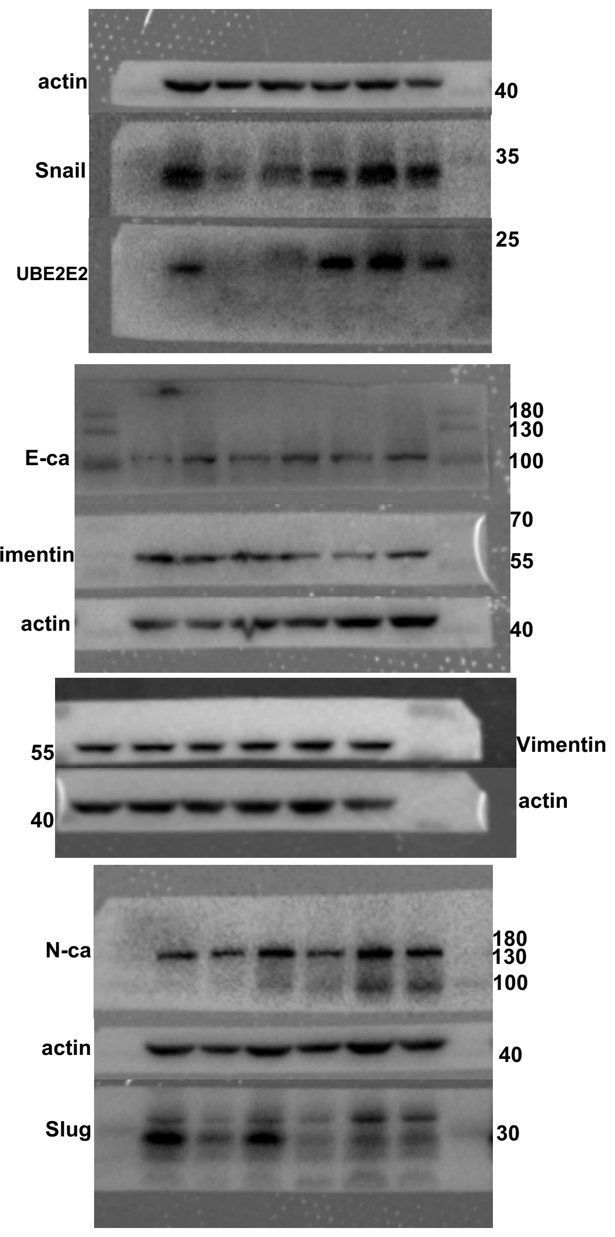

Fig.4A

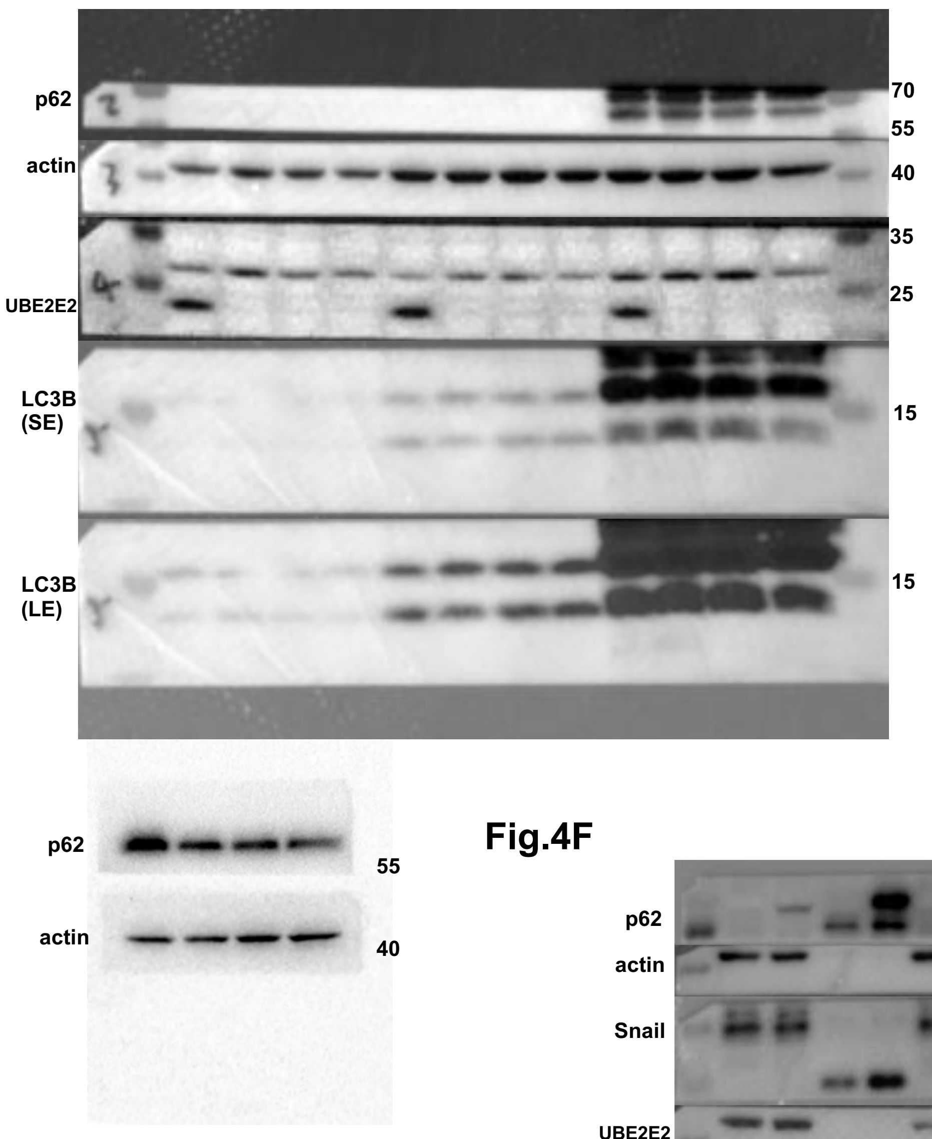

Fig.4B

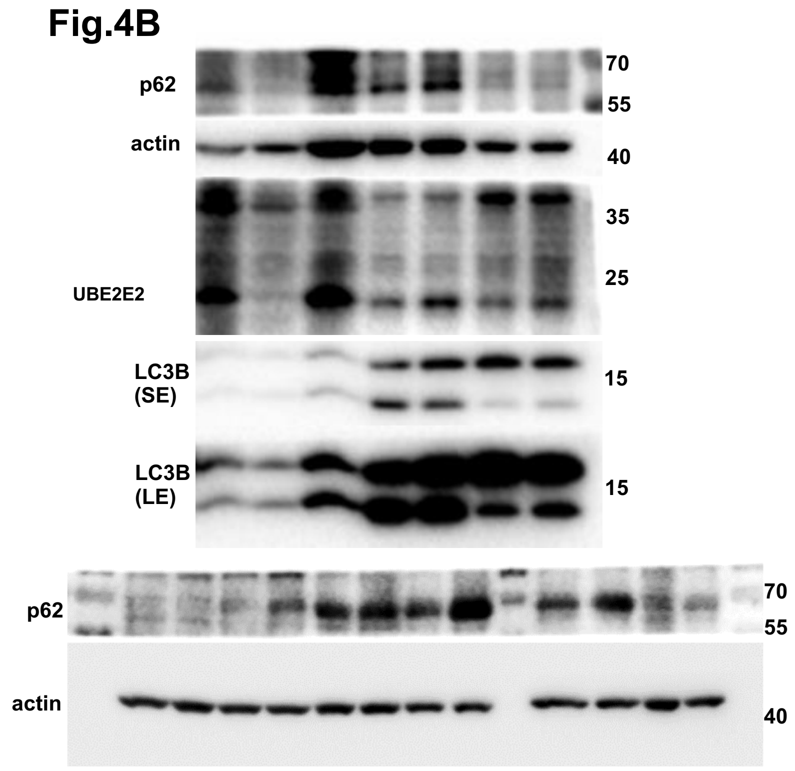

Fig.4D

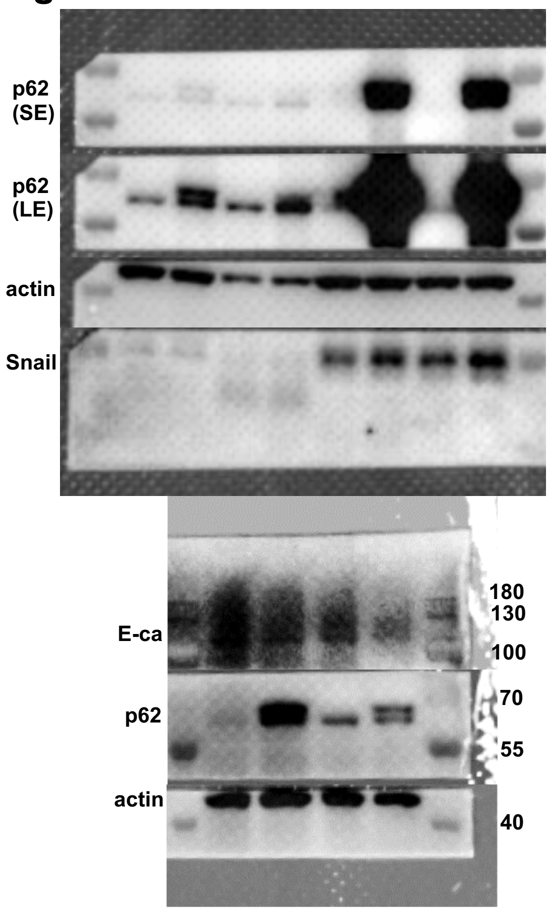

Fig.4F

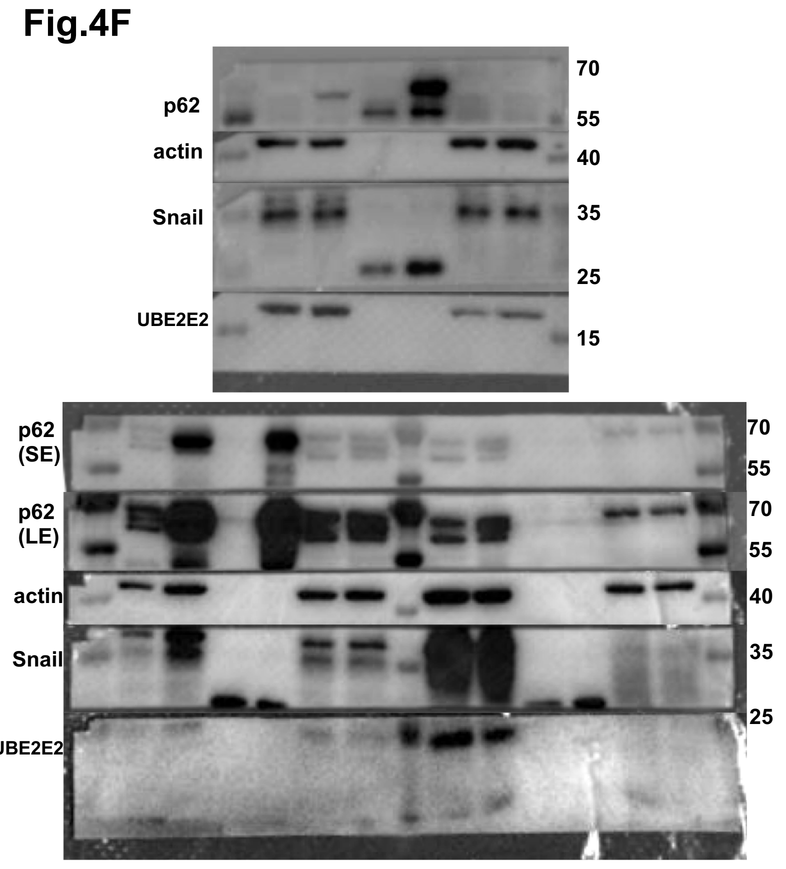

Fig.4G

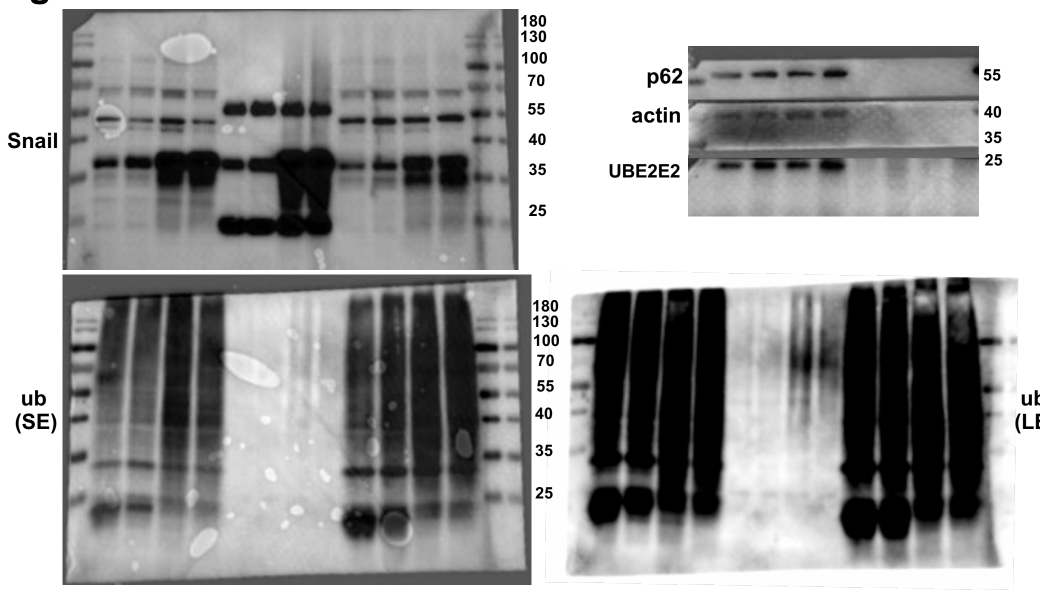

Fig.4E

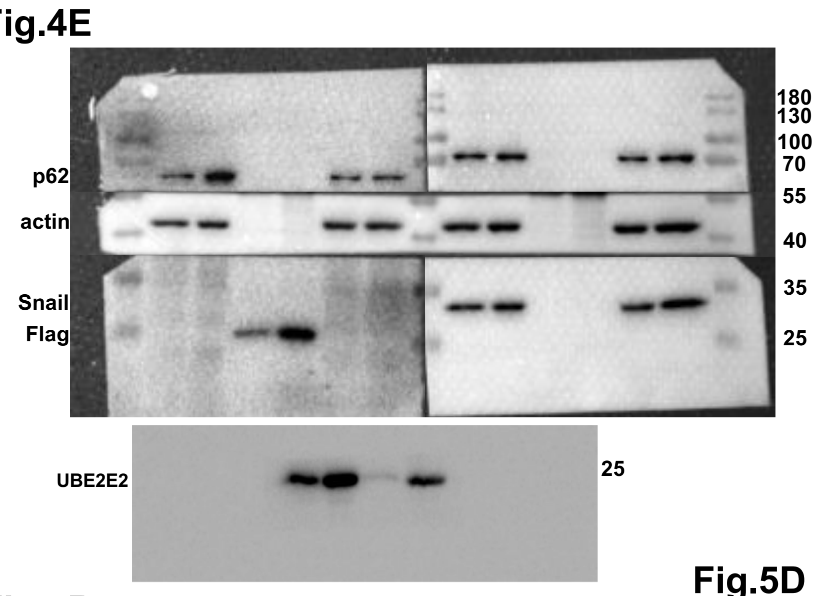

Fig.5A

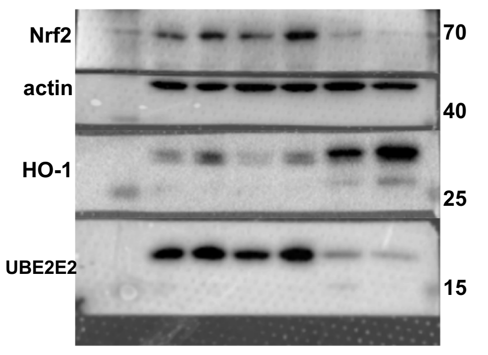

Fig.5B

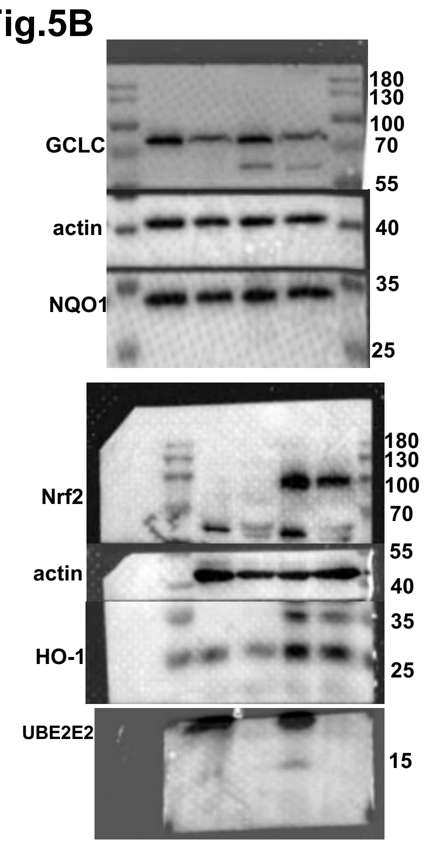

Fig.5D

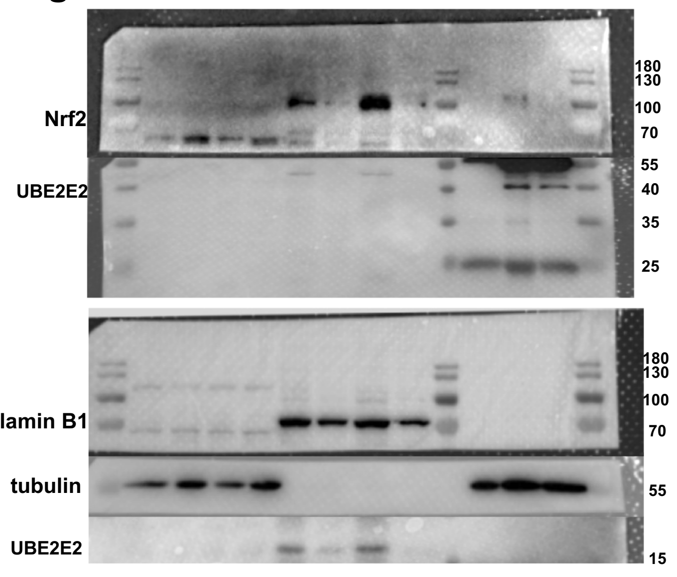

Fig.5E

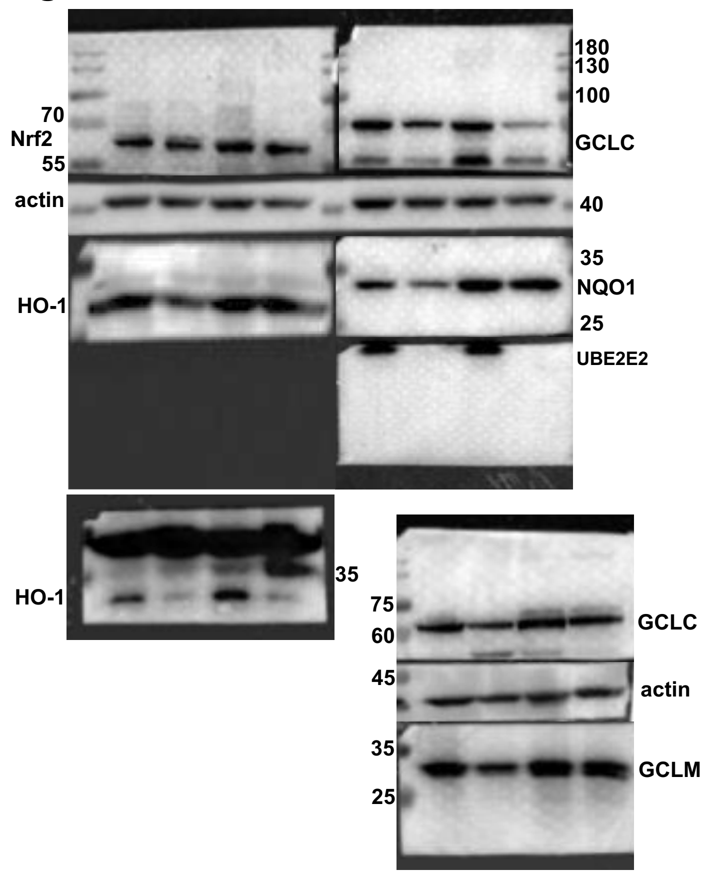

Fig.5H

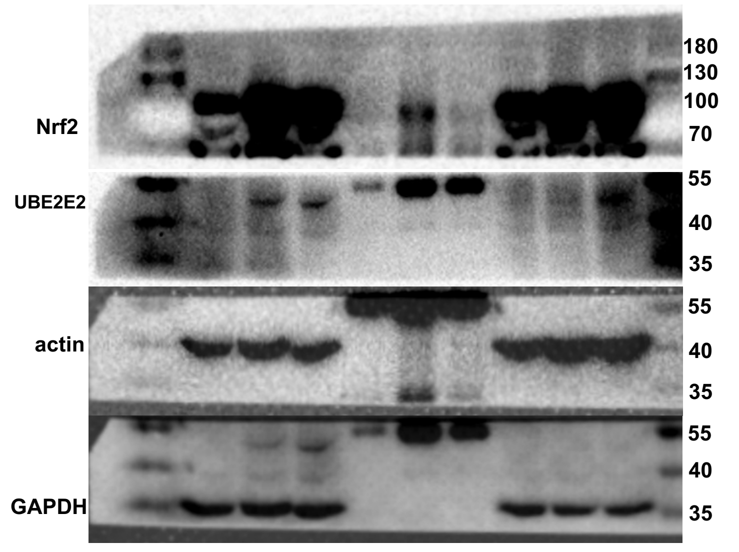

Fig.6E

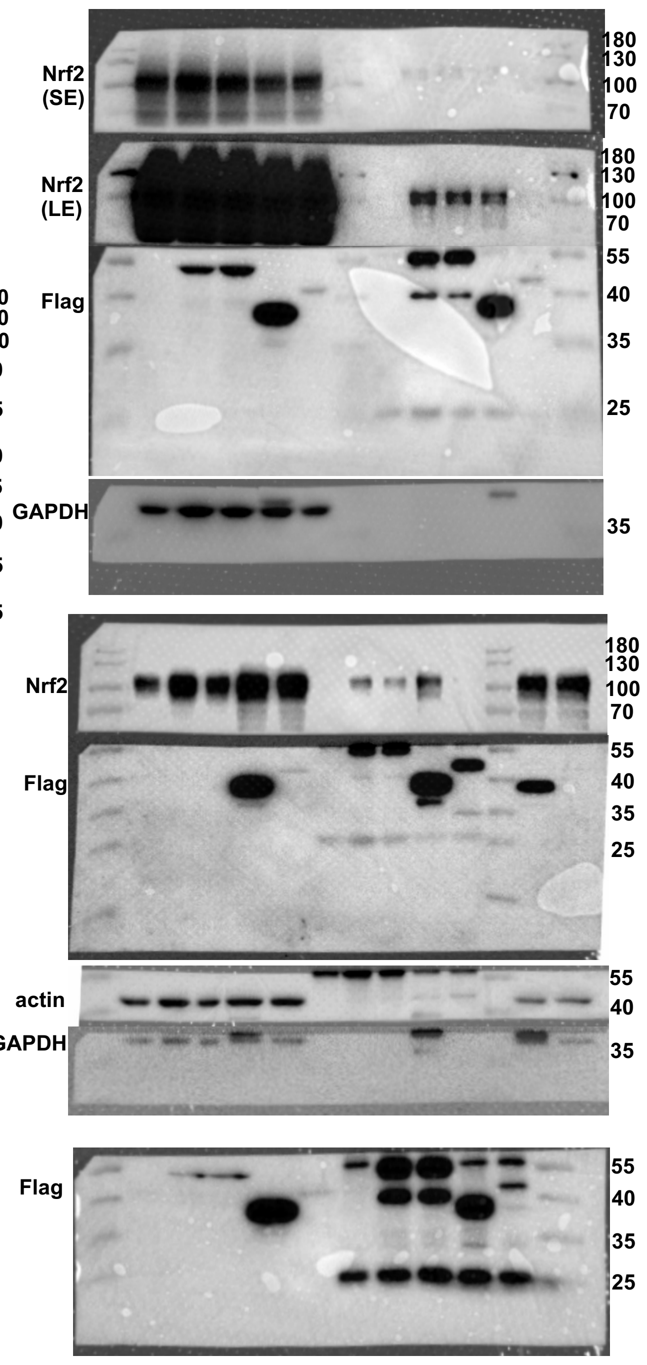

Fig.6D

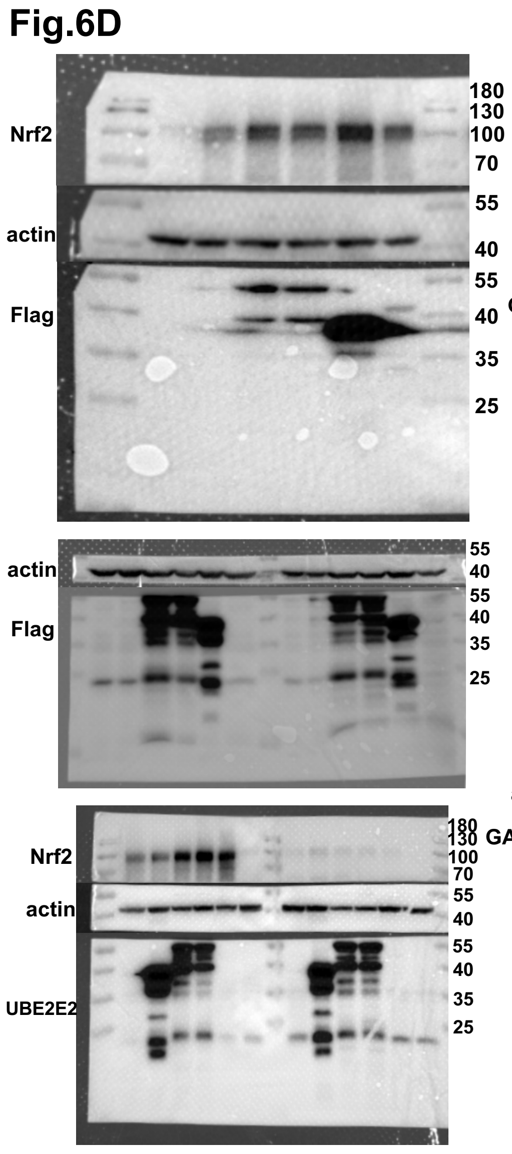

Fig.7D

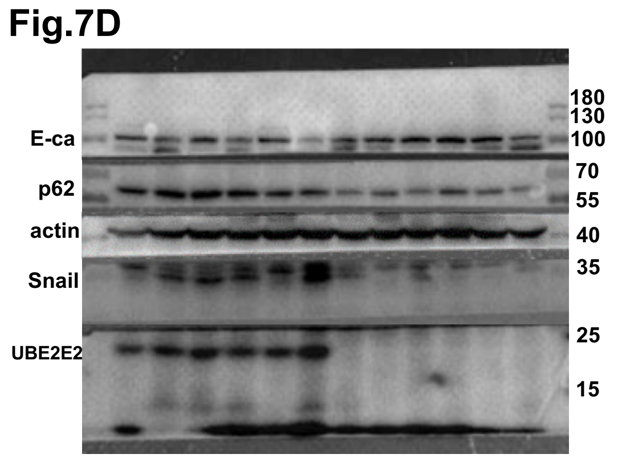

Fig.S2C

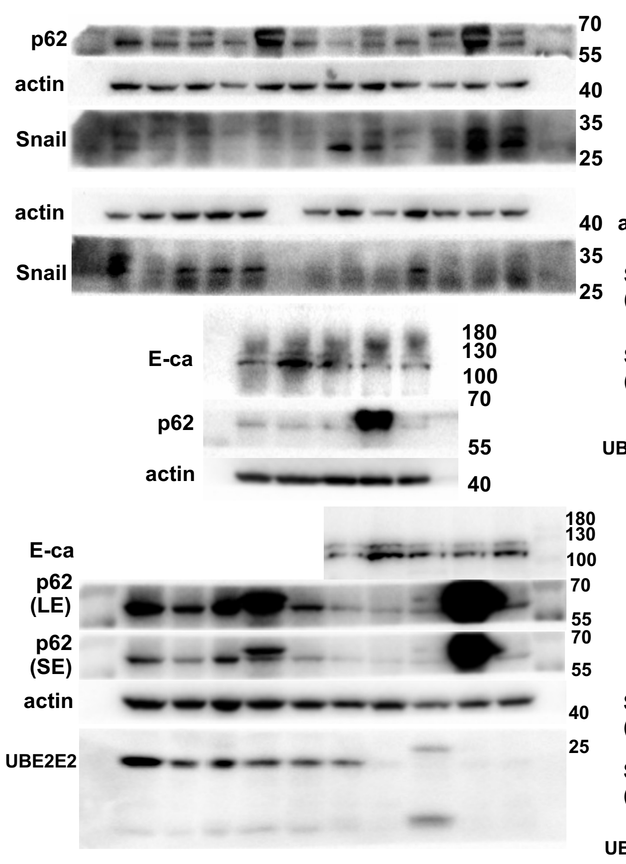

Fig.S3B

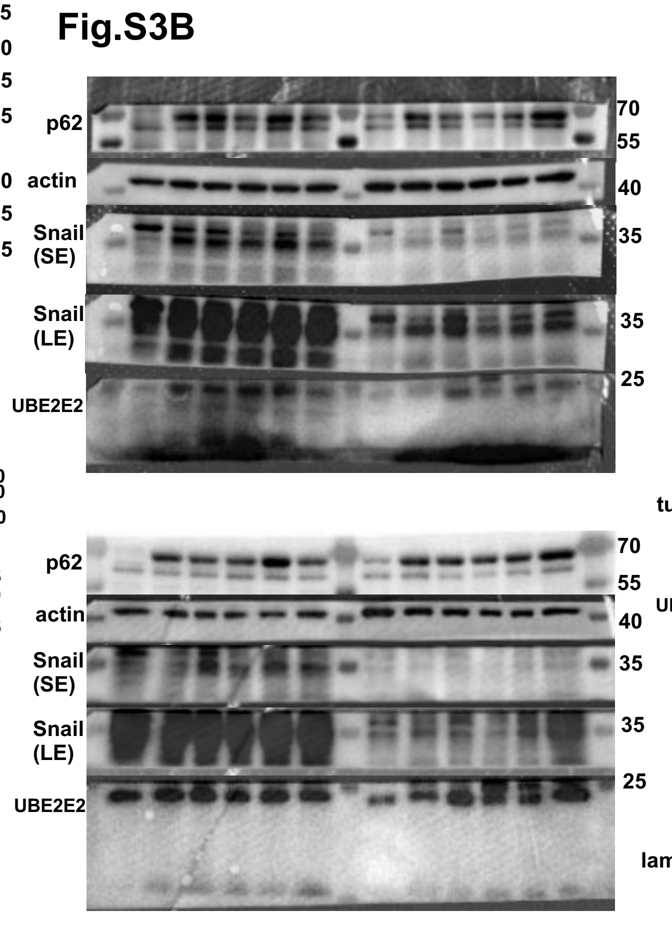

Fig.S3D

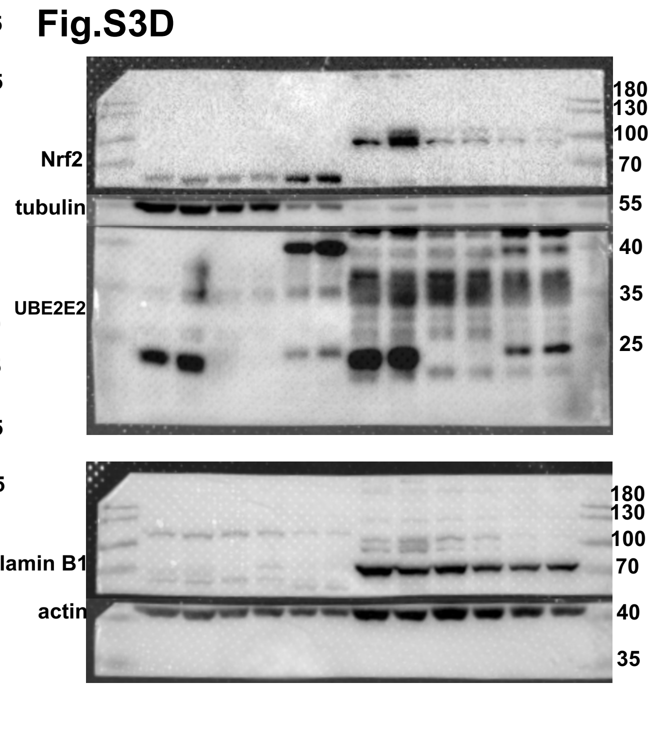

Supplement: Supplementary file 3 — original data files [file 41419_2023_5636_MOESM3_ESM.pdf]
